# Enzymatic and structural characterization of HAD5, an essential phosphomannomutase of malaria-causing parasites

**DOI:** 10.1016/j.jbc.2021.101550

**Published:** 2021-12-29

**Authors:** Philip M. Frasse, Justin J. Miller, Alexander J. Polino, Ebrahim Soleimani, Jian-She Zhu, David L. Jakeman, Joseph M. Jez, Daniel E. Goldberg, Audrey R. Odom John

**Affiliations:** 1 Division of Infectious Diseases, Departments of Medicine and Molecular Microbiology, Washington University School of Medicine, St. Louis, Missouri, USA; 2 Department of Biology, Washington University in St. Louis, St. Louis, Missouri, USA; 3 College of Pharmacy, Dalhousie University, Halifax, Nova Scotia, Canada; 4 Department of Chemistry, Razi University, Kermanshah, Iran; 5 Department of Chemistry, Dalhousie University, Halifax, Nova Scotia, Canada; 6 Division of Infectious Diseases, Department of Pediatrics, Children’s Hospital of Philadelphia, University of Pennsylvania, Philadelphia, Pennsylvania, USA

**Keywords:** malaria, parasite, glycosylphosphatidylinositol (GPI) anchor, carbohydrate metabolism, drug development, haloacid dehalogenase (HAD), phosphomannomutase, crystal structure, aTc, anhydrotetracycline, E, ethanolamine, E64, epoxysuccinyl-L-leucylamido(4-guanidino)butane, FSM, fosmidomycin, G-1,6-P, glucose-1,6-bisphosphate, G1P, glucose 1-phosphate, G6P, glucose 6-phosphate, GlcN, glucosamine, GPI, glycosylphosphatidylinositol, GPI-AP, GPI-anchored protein, HAD, haloacid dehalogenase, Ins, inositol, M1P, mannose 1-phosphate, M6P, mannose 6-phosphate, Man, mannose, ManN, mannosamine, MSP, merozoite surface protein, PGM, phosphoglucomutase, PGP, phosphoglycolate phosphatase, PMM, phosphomannomutase

## Abstract

The malaria-causing parasite *Plasmodium falciparum* is responsible for over 200 million infections and 400,000 deaths per year. At multiple stages during its complex life cycle, *P. falciparum* expresses several essential proteins tethered to its surface by glycosylphosphatidylinositol (GPI) anchors, which are critical for biological processes such as parasite egress and reinvasion of host red blood cells. Targeting this pathway therapeutically has the potential to broadly impact parasite development across several life stages. Here, we characterize an upstream component of parasite GPI anchor biosynthesis, the putative phosphomannomutase (PMM) (EC 5.4.2.8), HAD5 (PF3D7_1017400). We confirmed the PMM and phosphoglucomutase activities of purified recombinant HAD5 by developing novel linked enzyme biochemical assays. By regulating the expression of HAD5 in transgenic parasites with a TetR-DOZI-inducible knockdown system, we demonstrated that HAD5 is required for malaria parasite egress and erythrocyte reinvasion, and we assessed the role of HAD5 in GPI anchor synthesis by autoradiography of radiolabeled glucosamine and thin layer chromatography. Finally, we determined the three-dimensional X-ray crystal structure of HAD5 and identified a substrate analog that specifically inhibits HAD5 compared to orthologous human PMMs in a time-dependent manner. These findings demonstrate that the GPI anchor biosynthesis pathway is exceptionally sensitive to inhibition in parasites and that HAD5 has potential as a specific, multistage antimalarial target.

Malaria remains an enormous global health burden for most of the world, resulting in over 200 million infections and 400,000 deaths every year, the majority of which are in children under the age of 5 years (https://www.who.int/publications/i/item/9789240015791, accessed May 14, 2021). One of the primary barriers to effective malaria treatment and control is the emergence of resistance to all approved antimalarial chemotherapeutics (1, 2), prompting an urgent call for the development of new therapies and the identification of novel drug targets. Malaria is caused by apicomplexan parasites of the genus *Plasmodium*, primarily the species *Plasmodium falciparum*. *P. falciparum* has a complex life cycle, in which parasites develop in a mosquito host, are deposited into a human during a mosquito blood meal, and migrate to the liver where they infect hepatocytes. Parasites are released from the liver into the bloodstream and begin an asexual cycle of replication within red blood cells (RBCs), occasionally branching off into sexual-stage gametocytes that can be taken up by mosquitoes to start the cycle anew (3).

This complex, multihost life cycle has stymied efforts to develop therapeutics and vaccines to eradicate this disease, as it has been difficult to identify effective vaccine and therapeutic targets that span multiple life stages. In recent years, the push for new therapeutics and vaccines has focused on a strategy of developing transmission-blocking vaccines and therapies, which impair the development or viability of gametocytes or target the mosquito vector itself, thus preventing vector-borne transmission (4, 5, 6). Our goal is therefore to identify novel targets for antimalarial therapeutics that are not only essential for intraerythrocytic growth of the parasite but also essential for sexual-stage and/or mosquito-stage parasites, indicating their potential in transmission-blocking strategies.

Malaria parasites are highly metabolically active (7, 8), and several known antimalarials target unique and essential metabolic processes in the parasites (9, 10, 11, 12, 13). Metabolic enzymes have great potential for chemical inhibition, as substrate analogs can be rationally designed and developed as potential inhibitors, making these enzymes well suited as “druggable” targets (14). We therefore sought a metabolic enzyme that is expressed and essential during multiple life stages of *P. falciparum*. These constraints narrowed our search to the upstream steps of glycosylphosphatidylinositol (GPI) anchor synthesis.

GPI anchors are an essential component of all life stages of *P. falciparum*. In intraerythrocytic parasites, these glycolipid anchors tether several essential proteins to the parasite plasma membrane prior to RBC egress and reinvasion (15). Most abundant among these GPI-anchored proteins (GPI-APs) is merozoite surface protein 1 (MSP1) (15, 16, 17). Proper MSP1 localization and processing are necessary for parasites to egress from the erythrocyte, and mature MSP1 anchored to the surface of free merozoites facilitates the binding and invasion of new RBCs. In the absence of the GPI-anchoring C terminus of MSP1, parasites are defective in their ability to egress (18), and antibodies developed against MSP1 prevent merozoite reinvasion (19). Several other GPI-APs are also involved in parasite egress and invasion, including other MSPs and rhoptry-associated membrane antigen (15, 17, 20). GPI-APs are also expressed in other life stages of *P. falciparum*. Gamete-stage and ookinete-stage GPI-APs include Pfs25 and Pfs230, which are considered as possible vaccine candidates (21, 22, 23, 24), while circumsporozoite protein, the critical antigen of the RTS,S and R21 vaccines, is an essential GPI-AP of the sporozoite stages (25, 26, 27, 28, 29). Thus, it is clear that successful targeting of GPI anchor biosynthesis would not only effectively treat symptomatic blood-stage infection but may also block transmission of the parasites at multiple stages.

One enzyme of the GPI anchor biosynthesis pathway that is yet to be characterized in *P. falciparum* is the putative phosphomannomutase (PMM), HAD5 (PMM; PF3D7_1017400). PMMs are responsible for the conversion of mannose 6-phosphate (M6P) to mannose 1-phosphate (M1P), the precursor to GDP-mannose. GDP-mannose is then converted to dolichol-p-mannose, which is the building block for incorporating mannose into glycolipids. In asexual *P. falciparum*, the dominant mannosylated glycolipids are GPI anchors (30), as *N*-glycans in these parasites only contain *N*-acetyl glucosamine (31, 32, 33); therefore, targeting of mannose metabolism is predicted to specifically inhibit GPI anchor synthesis. HAD5 is also predicted to be essential (34), and transcriptomic studies show its expression during the blood stage (35, 36) and sexual stages (37), making it a potential multistage antimalarial drug target. In this study, we characterize the putative PMM of *P. falciparum*, HAD5, demonstrating its essentiality for parasite growth and its potential for specific targeting by future antimalarial therapies.

## Results

### HAD5 is a PMM

**Figure 1:**
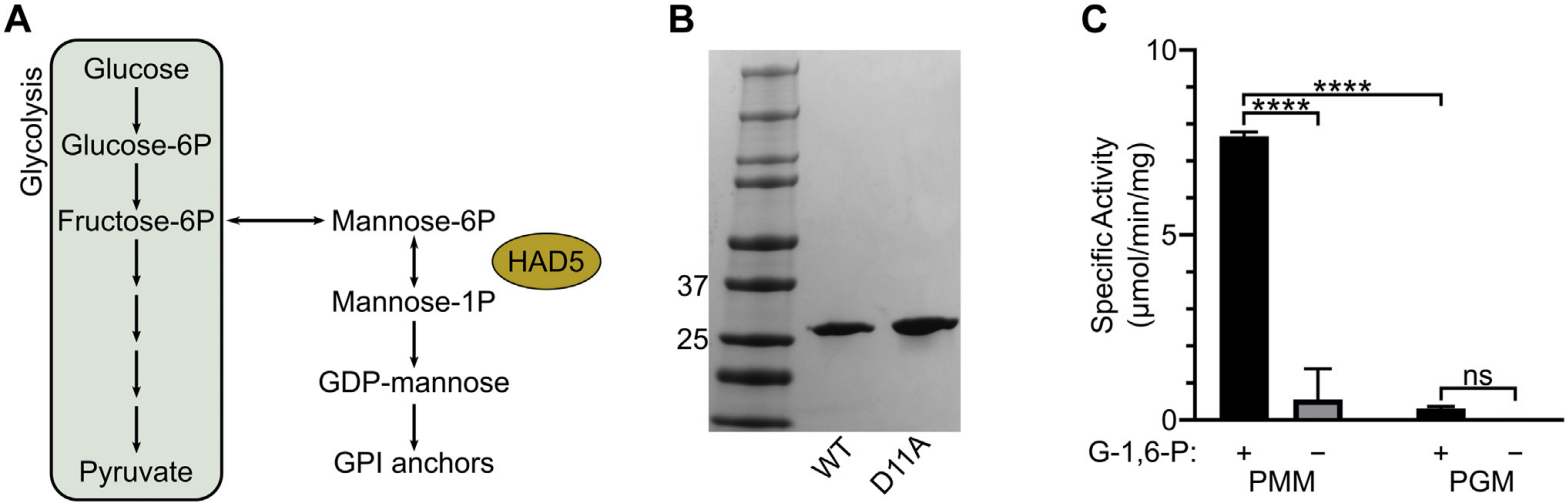
**HAD5 is a bifunctional phosphomannomutase/phosphoglucomutase.***A*, schematic of phosphomannomutases’ role in metabolism. Phosphomannomutases like HAD5 interconvert mannose 6-phosphate and mannose 1-phosphate, providing the latter for downstream glycolipid production and synthesis of GPI anchors in *P. falciparum*. *B*, SDS-PAGE gel of recombinant WT HAD5 and a catalytically inactive mutant (D11A). *C*, displayed are the mean ± SEM of HAD5 activity across three independent trials, with D11A activity subtracted as background. *p*-values were determined using an ordinary two-way ANOVA (Tukey's test for multiple comparisons, α = 0.05). ∗∗∗∗*p* < 0.0001, ns = not significant. G-1,6-P, glucose-1,6-bisphosphate; GPI, glycosylphosphatidylinositol; PGM, phosphoglucomutase assay; PMM, phosphomannomutase assay.

HAD5 (PF3D7_1017400) has been annotated as a putative PMM through homology to other known PMMs. PMMs are responsible for interconverting M6P and M1P to generate M1P for downstream glycolipid production, most notably GPI anchors in *P. falciparum* (Fig. 1*A*) (38, 39, 40). To determine the biochemical function of HAD5, we purified recombinant HAD5 (Fig. 1*B*) and examined its hexose phosphate mutase activities (Figs. 1*C* and S1). We found that HAD5 was active in the PMM assay (0.56 ± 0.48 μmol/min/mg), with even more robust activity (7.67 ± 0.06 μmol/min/mg) upon addition of the known cofactor glucose-1,6-bisphosphate (G-1,6-P) (40), which is comparable to the specific activity of other PMMs (41, 42). Although turnover number (k_cat_) was much lower in our assays than that of other assays for PMM activity (43, 44), it should be noted that the majority of the published work on PMM activity has investigated the reverse PMM reaction, converting M1P to M6P, at 37 °C. It is therefore difficult to directly compare the reaction rate of our forward reaction assay at room temperature. Furthermore, as has been seen for other PMMs (42, 45), HAD5 exhibits some promiscuity in its substrate preference, as it also displays phosphoglucomutase (PGM) activity at 0.31 ± 0.03 μmol/min/mg. Comparison of the catalytic efficiencies of HAD5 toward phosphomannose and phosphoglucose suggests that PMM activity is the dominant enzymatic function of HAD5, with a roughly 4-fold higher catalytic efficiency (k_cat_/K_m_) compared to its PGM activity (Table 1). HAD5 activity against other phosphosugars, or using other bisphosphate activators, was not assessed.

**Table 1:** Phosphomannomutase and phosphoglucomutase activity of HAD5

Enzyme assay	*k*_*cat*_ (s^−1^)	*K*_*m*_ (μM)	*k*_*cat*_*/K*_*m*_ (M^−1^ s^−1^)
PMM	1.7 × 10^−4^ ± 1.9 × 10^−5^	32 ± 3.6	5.4
PGM	7.8 × 10^−6^ ± 1.2 × 10^−6^	5.2 ± 1.4	1.5

Displayed are the mean ± SEM of three independent trials for the kinetic parameters of HAD5 converting M6P to M1P (PMM) or G1P to G6P (PGM).

Abbreviations: G1P, glucose 1-phosphate; G6P, glucose 6-phosphate; PGM, phosphoglucomutase; PMM, phosphomannomutase.

### HAD5 is essential for intraerythrocytic parasite growth

**Figure 2:**
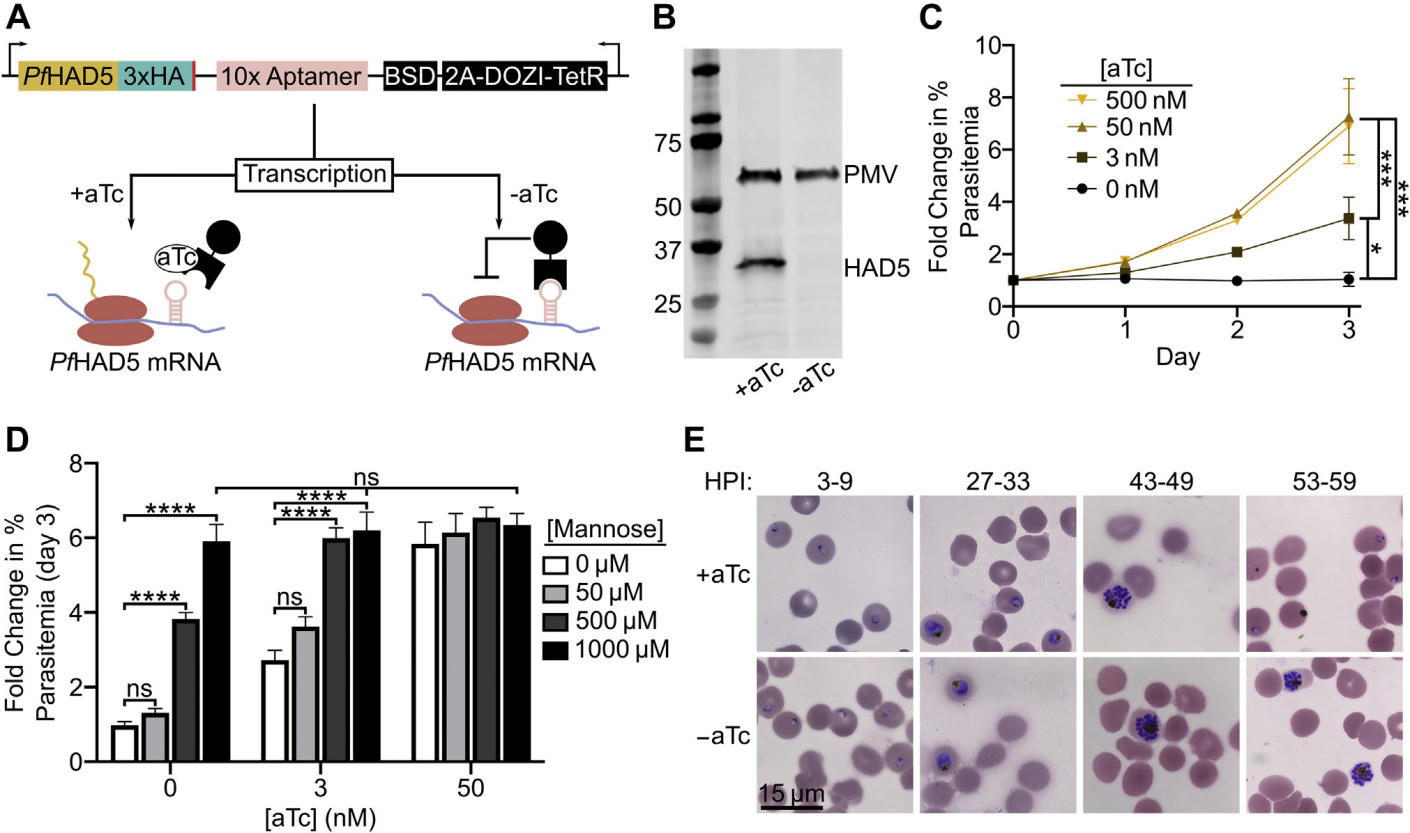
**HAD5 is essential for intraerythrocytic parasite growth.***A*, schematic of the regulatable knockdown system introduced at the native locus of *Pf*HAD5 (46, 47). *B*, Western blot of transgenic HAD5^KD^ parasite lysate ±aTc, using α-HA to detect HAD5 and α-Plasmepsin V (PMV) as a loading control. Removal of aTc results in successful knockdown of HAD5. Approximate expected protein masses: PMV, 51 kDa; *Pf*HAD5-3xHA, 32 kDa. *C*, fold change in parasitemia over time of HAD5^KD^ parasites grown in varying concentrations of aTc. Data represent mean ± SEM of three independent experiments with technical duplicates. Significance was determined by one-way ANOVA with Fisher's LSD; ∗*p* = 0.03; ∗∗∗*p* < 0.001. *D*, fold change in parasitemia of HAD5^KD^ parasites grown in varying aTc concentrations with D-mannose rescue. Significance was determined by ordinary two-way ANOVA with Tukey's correction for multiple comparisons. ∗∗∗∗*p* < 0.0001, ns = not significant. Data represent mean ± SEM of three independent experiments with technical duplicates. *E*, bright-field images of Giemsa-stained thin-smear synchronized parasites over time. aTc, anhydrotetracycline; HPI, hours postinvasion.

To assess the essentiality of HAD5 in intraerythrocytic parasite stages, we used a previously described conditional knockdown system in cultured asexual *P. falciparum* (46, 47) (Fig. 2*A*). We placed a Tet repressor-binding aptamer array at the 3′-end and 5′-end of the endogenous HAD5 locus by CRISPR/Cas9-mediated integration. In parasite integrants, the presence of anhydrotetracycline (aTc) promotes HAD5 translation, while washing out aTc leads to inhibition of translation, and we term this conditional knockdown strain “HAD5^KD^”. Immunoblotting confirms substantial reduction in cellular abundance of HAD5 in the absence of aTc (Fig. 2*B*). In HAD5^KD^ parasites, 0 nM aTc conditions led to an absence of growth, whereas addition of aTc promoted growth in a dose-dependent manner (Fig. 2*C*), indicating that HAD5 is essential for asexual growth of *P. falciparum*.

Because phosphosugar mutases often utilize more than one substrate, we examined whether the PMM activity of HAD5 was responsible for its essential function in asexual parasites. Attempts to chemically rescue parasite growth with hexose phosphates such as M6P and M1P were unsuccessful (Fig. S2*A*), as was expected because of the impermeability of the erythrocyte and parasite membranes to such highly charged compounds. However, we found that simple chemical supplementation of the media with D-mannose was sufficient to rescue growth when HAD5 expression is reduced. This indicates that the primary mechanism of death in these parasites is due to defects in mannose metabolism (Figs. 2*D* and S2*B*). Notably, while artificially elevated concentrations of D-mannose completely rescued parasite growth, a physiologically relevant concentration of 50 μM [equivalent to that of human serum (48)] did not provide a statistically significant rescue of parasite growth.

### HAD5 is required for parasite egress and invasion

GPI anchors, which contain mannose (15, 17, 30), are required for egress of the malaria parasite from the infected host cell, as well as reinvasion of merozoites (47, 49, 50, 51, 52, 53, 54, 55, 56). Because of its essential role in mannose metabolism, we hypothesized that HAD5 may be required for efficient egress and invasion. To evaluate this possibility, we washed out aTc at the beginning of the life cycle in synchronized parasites. Parasites grown in −aTc conditions over the course of one life cycle developed morphologically normally through the majority of life cycle stages, including the development of multinucleated schizonts. While +aTc conditions allowed parasites to continue into the next life cycle and form newly reinvaded “ring”-stage parasites, −aTc parasites were arrested in late schizogony (Fig. 2*E*), which suggests a defect in parasite egress when HAD5 expression is knocked down. Notably, when HAD5 expression is reduced, parasites retain normal mature schizont architecture by transmission electron microscopy, indicating that neither gross developmental defects nor structural aberrations prevent parasites from egressing (Fig. S3).

To examine whether HAD5 is required for invasion, as well as egress, we examined the reinvasion capacity of HAD5^KD^ parasites. Segmented schizont-enriched cultures of HAD5^KD^ ±aTc were mechanically lysed, and the freed merozoites were allowed to reinvade fresh RBCs. Under these conditions, knockdown parasites were unable to reinvade new host cells (Fig. S4). Thus, the knockdown of HAD5 confers defects to parasite biology that prevent both egress and reinvasion of the parasites.

### HAD5 knockdown disrupts GPI anchor synthesis

**Figure 3:**
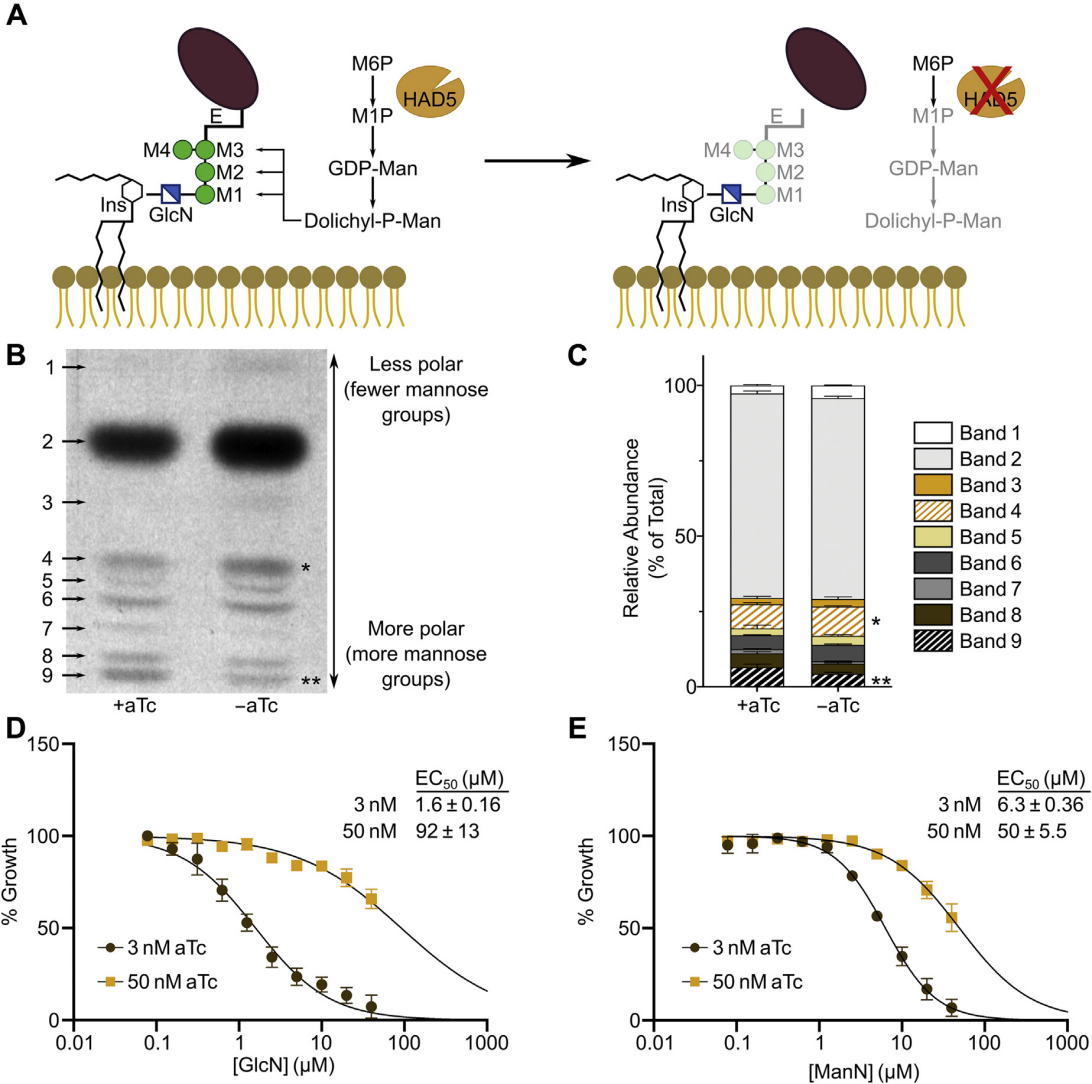
**Knockdown of HAD5 disrupts GPI anchor biosynthesis.***A*, model of the predicted effect that knocking down HAD5 will have on GPI anchor precursor synthesis and subsequent anchoring of GPI-APs. *B*, representative autoradiography film of GPI anchor precursors from [^3^H]GlcN-labeled parasites. Bands toward the top migrated farthest on a silica TLC plate, indicating they are less polar and less mannosylated. *C*, the radiographic signal was quantified and is represented as proportions of total signal. Band numbers indicate the corresponding band from *A*. Shown are the mean and SEM of three independent experiments, analyzed by ordinary two-way ANOVA with Fisher's LSD test. ∗*p* = 0.038, ∗∗*p* = 0.008. *D* and *E*, dose–response curve of parasite growth in the presence of GlcN (*D*) or mannosamine (ManN; *E*). Data represent the means and SEM of three independent experiments with technical replicates. Aps, anchored proteins; E, ethanolamine; GlcN, glucosamine; GPI, glycosylphosphatidylinositol; Ins, inositol; M, mannose; M1P, mannose 1-phosphate; M6P, mannose 6-phosphate.

Mannose metabolism is linked to parasite egress through biosynthesis of GPI anchors (57). In *P. falciparum*, GPI anchors are synthesized through addition of one glucosamine (GlcN) and 3 to 4 mannose residues to a phosphatidylinositol backbone. These mannose residues are derived from the product of PMM, M1P, which is converted to GDP-mannose and subsequently to dolichol-phosphate mannose, the direct mannose donor to GPI anchors (Fig. 3*A*) (58). Several GPI-APs contribute to egress and invasion of parasites (15, 18, 20). We therefore hypothesized that reduced HAD5 expression leads to loss of PMM activity and causes parasite death by disruption of GPI anchor biosynthesis. To directly evaluate the effect of HAD5 knockdown on GPI anchor biosynthesis, we labeled mid- to late-trophozoite parasites with [^3^H]-GlcN and extracted GPI precursors as previously described (17, 57, 59, 60). HAD5^KD^ parasites grown in +aTc conditions had the expected repertoire of GPI anchor precursors (Fig. 3, *B* and *C*). A variety of precursors are observed, with earlier, less polar species (with fewer mannose groups) migrating further than more polar, highly mannosylated species. When HAD5 expression is reduced, there is a relative accumulation of the earlier precursors, as well as a reduced production of fully mature, highly mannosylated precursors (Fig. 3*B*), indicating a defect in GPI anchor biosynthesis. In particular, there was a significant reduction of the highly polar band 9 and significant buildup of less polar band 4 when HAD5 expression was reduced (Fig. 3*C*). Intriguingly, despite the substantial knockdown of HAD5 and the complete loss of growth in these parasites, the abundance of many mannosylated precursors is unchanged and highly mannosylated GPI precursors are still observed, suggesting that this biosynthetic pathway is not completely ablated.

To confirm the role of HAD5 in GPI biosynthesis, we deployed two established chemical tools, mannosamine (ManN) and GlcN, which impair the growth of *P. falciparum* through inhibition of GPI anchor biosynthesis (61, 62). We expected that if HAD5 knockdown disrupts GPI anchor biosynthesis, parasites should be hypersensitized to ManN and GlcN treatment. Indeed, when the expression of HAD5 is reduced with an intermediate concentration of aTc (3 nM) that still permits modest asexual growth, knockdown parasites yielded a marked shift in EC_50_ (Fig. 3, *D* and *E*). These results further implicate HAD5 in the production of GPI anchors in *P. falciparum*.

### HAD5-dependent GPI anchor synthesis enables proper anchoring of MSP1

**Figure 4:**
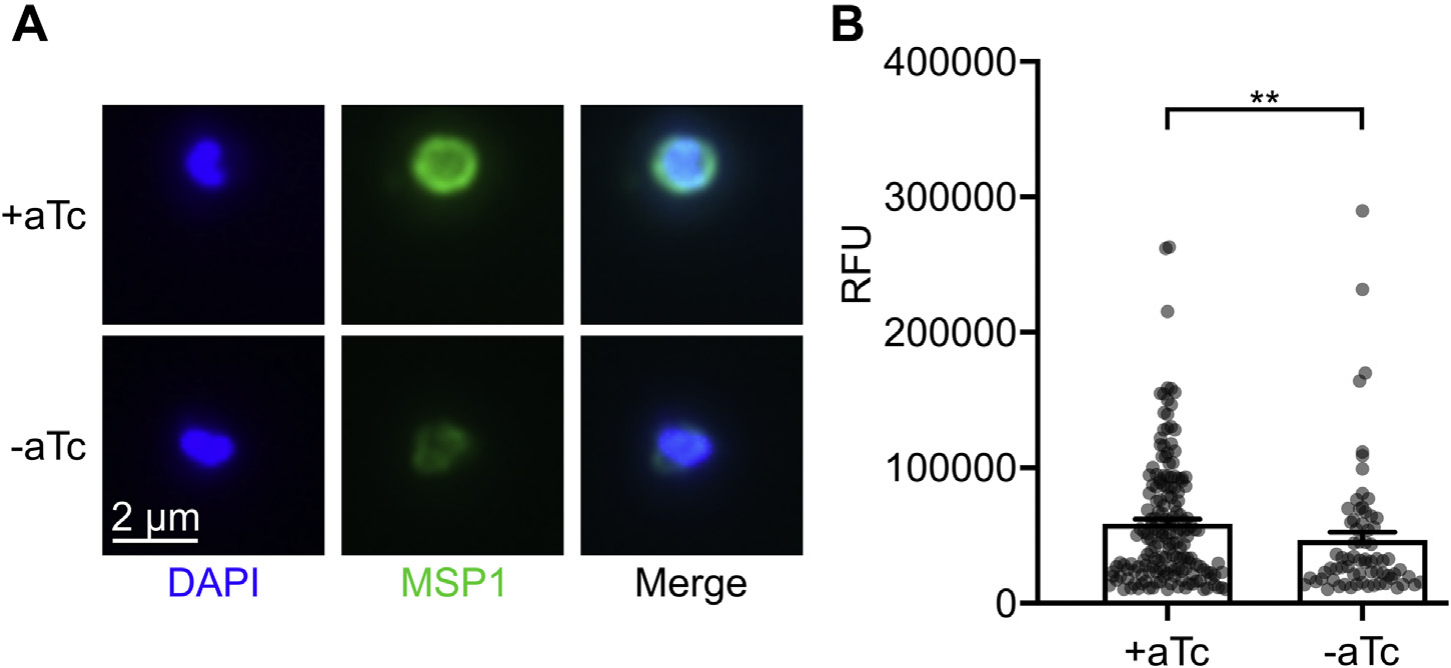
**Knockdown of HAD5 diminishes membrane anchoring of the egress and invasion protein MSP1.***A*, representative immunofluorescent images of mechanically freed merozoites that were grown in ±aTc conditions and schizont enriched by E64 treatment. *B*, quantification of MSP1 signal from *A*. Data points represent three independent experiments, each with >25 observed merozoites, removing those under a threshold of 10,000 RFU. Bar graphs represent the mean ± SEM of all data points. Statistics were performed by Mann–Whitney test. ∗∗*p* = 0.008. aTc, anhydrotetracycline; E64, epoxysuccinyl-L-leucylamido(4-guanidino)butane; MSP1, merozoite surface protein 1.

Reduced GPI anchor biosynthesis in malaria parasites is expected to impact the localization and function of a number of essential GPI-anchored parasite proteins. While several GPI-APs have been characterized in *P. falciparum* intraerythrocytic stages, the most abundant is MSP1 (15, 16). MSP1 must be targeted and anchored through GPIs and proteolytically processed for schizont-stage parasites to egress from the erythrocyte, and the MSP1 complex is also critical for binding and reinvading new RBCs (18, 63). For this reason, we investigated whether HAD5-dependent GPI anchor synthesis is required for localization and anchoring of MSP1. We expected that, when the pathway is intact, MSP1 is successfully anchored to the parasite plasma membrane. In contrast, when HAD5 expression is knocked down, GPI anchors will fail to fully incorporate mannose, and GPI-APs, including MSP1, will remain untethered to the membrane (Fig. 3*A*). To evaluate this effect, we used immunofluorescence to detect the localization of MSP1. When schizonts grown in ±aTc conditions were mechanically lysed and the resultant merozoites were imaged, there was a modest but significant decrease in MSP1 signal surrounding the daughter merozoites when HAD5 expression is reduced (Fig. 4, *A* and *B*), indicating that MSP1 membrane attachment is diminished, causing it to diffuse away from the cell.

To independently confirm this finding, we partitioned lysate from early schizont-stage parasites into membranous and soluble fractions (64). Whole lysate and fractions were assessed by immunoblotting for MSP1, and the relative proportion of MSP1 in the membrane fraction compared to lysate was calculated. In +aTc conditions, a large proportion of MSP1 separates with the detergent-enriched phase, with some full-length MSP1 in the soluble phase. Upon HAD5 knockdown, there was a subtle reduction in the proportion of cellular MSP1 bound to the membrane (Fig. S5). This difference did not reach significance; however, substantial biological variability in MSP1 expression is present within merozoite populations, as indicated in Figure 4*B*, and post hoc power analysis of these data indicates that we were underpowered to observe a significant difference (power = 0.290) (65). Together with our analysis of GPI anchor biosynthesis in these parasites (Fig. 3), these data confirm some functional preservation of GPI biosynthesis when HAD5 expression is reduced.

### Disruption of mannose metabolism by HAD5 knockdown does not affect fosmidomycin sensitivity in parasites

HAD5 and other PMMs are part of the haloacid dehalogenase (HAD) superfamily of proteins (Interpro: IPR023214), a ubiquitous family of enzymes that primarily conduct phosphatase and phosphotransferase reactions (66). HAD5 is a member of subfamily IIB (IPR006379) of the HAD superfamily and has substantial sequence homology to other *P. falciparum* HAD proteins in this subfamily (HAD1 and HAD2) and the related subfamily IIA (IPR006357) protein, phosphoglycolate phosphatase (67). One notable commonality between these subfamily II *P. falciparum* HAD proteins is their effect on the parasite’s sensitivity to the antimalarial fosmidomycin (FSM), which is a well-validated inhibitor of the apicoplast methylerythritol phosphate pathway of isoprenoid biosynthesis (9). Mutations in either HAD1 (68) or HAD2 (69) render parasites resistant to FSM, and phosphoglycolate phosphatase knockout parasites are hypersensitive to FSM (70). To examine whether HAD5 also plays a role in FSM susceptibility, we assessed the FSM dose–response of parasites grown in saturating or intermediate aTc conditions. Unlike its close homologs, HAD5 knockdown had no effect on the FSM EC_50_ of parasites (Fig. S6).

### HAD5 is distinct from human PMMs and can be specifically inhibited

**Figure 5:**
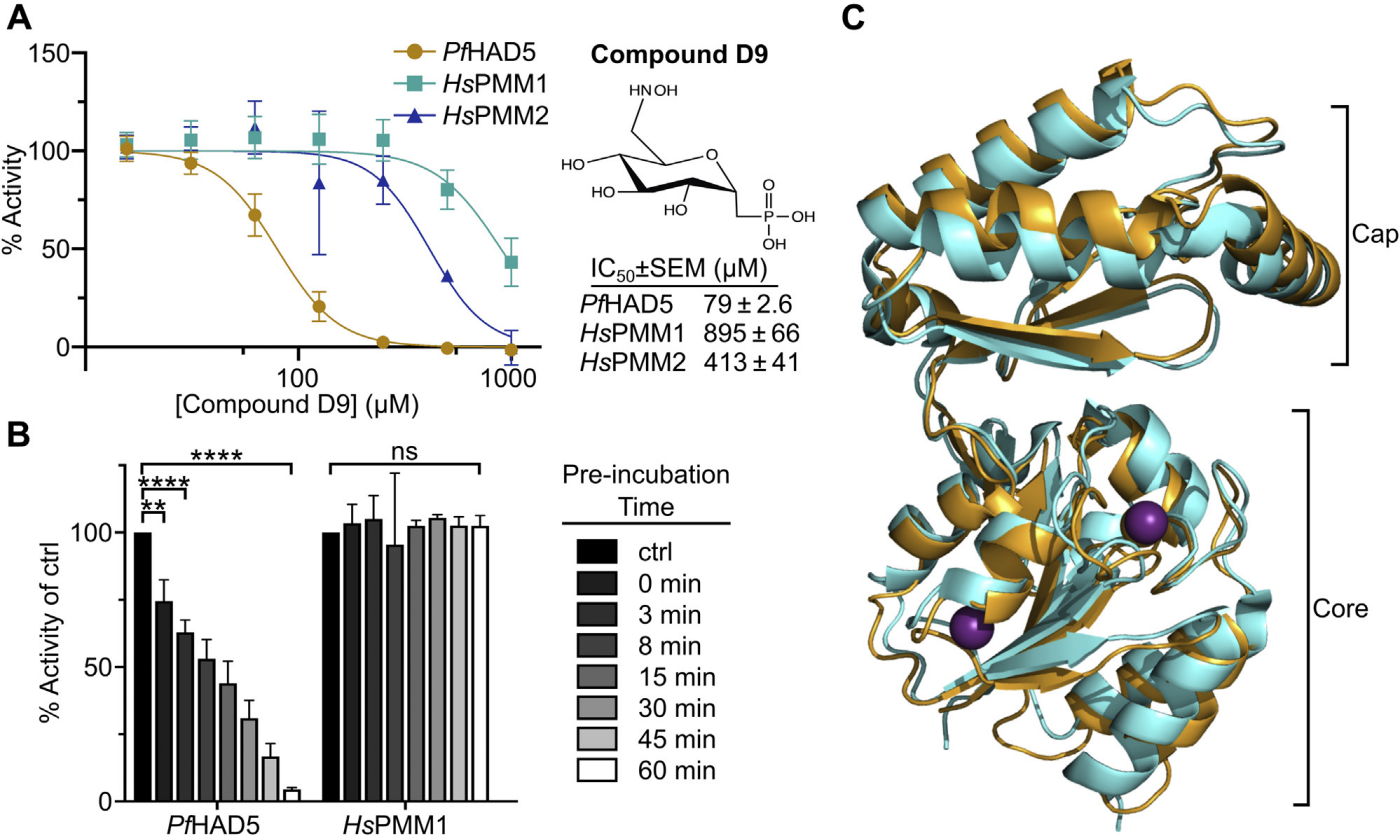
**HAD5 is sufficiently distinct from human PMMs to be specifically inhibited.***A*, dose–response curve of compound D9 against recombinant *Pf*HAD5, *Hs*PMM1, and *Hs*PMM2. Data represent the mean ± SEM of three independent experiments, each with technical replicates. *B*, activity of *Pf*HAD5 or *Hs*PMM1 was assayed after preincubating enzymes for the given time with 416 μM of D9 prior to adding the preincubation to the reaction mix (final [D9] = 50 μM). As a control (ctrl), enzymes were incubated with an equal volume of water for 60 min. Statistics were performed with an ordinary two-way ANOVA, using Dunnett's test for multiple comparisons. ∗∗*p* = 0.0046, ∗∗∗∗*p* < 0.0001, ns, not significant. *C*, 3.5 Å resolution crystal structure of PfHAD5 (*orange*) aligned to HsPMM1 (*cyan*; PDB 2FUC) with Mg^2+^ ions (*purple*). Indicated are the cap and core domains typical of HAD enzymes. HAD, haloacid dehalogenase; PMM, phosphomannomutase.

Previous studies have investigated egress and invasion as a promising target for antimalarial drug discovery, suggesting that HAD5 may likewise be of interest for antimalarial drug discovery (47, 49, 50, 51, 52); however, PMMs are found widely throughout nature, including two genes in the human genome, *Hs*PMM1 and *Hs*PMM2 (71, 72). We therefore evaluated the potential for selective inhibition of *P. falciparum* HAD5 over human PMM1 and PMM2 (Fig. S7*A*). Previous work has successfully demonstrated the use of substituted ketoheptoses and other phosphosugar analogs as inhibitors of microbial PMMs (73, 74), which we sought to replicate with HAD5. Using a panel of 11 phosphosugar analogs (Fig. S8*A*), we screened each compound for its ability to inhibit recombinant HAD5, PMM1, and PMM2. The majority of the compounds had negligible inhibition against all three enzymes (Fig. S8). Compound D9, however, inhibits purified recombinant HAD5 with a half-maximal inhibitory concentration of 79 ± 2.6 μM, severalfold more potently than the inhibition of either PMM1 or PMM2 (Fig. 5*A*). Moreover, we find dramatic time-dependent effects on the ability of D9 to inhibit HAD5, as preincubating HAD5 with D9 prior to assaying activity substantially increased D9 potency, such that a 60-min preincubation yielded HAD5 activity of only 4.5% of a vehicle-treated control (Fig. 5*B*). This effect was not seen for *Hs*PMM1, demonstrating that the potential to specifically inhibit HAD5 may be even greater under ideal binding conditions. As expected, given its poor drug-like characteristics (and likely inadequate cellular permeability), compound D9 did not impair the growth of asexual *P. falciparum* at concentrations up to 100 μM (Fig. S9*A*). The selective inhibition of HAD5 by D9 is an important proof-of-concept that distinct structural features of HAD5 may be harnessed for parasite-specific inhibitor development.

To uncover the structural basis for HAD5-specific inhibition, we solved the X-ray crystal structure of HAD5 to 3.5 Å resolution. Data collection and refinement statistics are summarized in Table 2. Like other known PMMs and members of the HAD superfamily (66, 75), HAD5 comprises two domains with an overall fold similar to that of *Hs*PMM1 (Fig. 5*C*). There were no large structural differences in the overall three-dimensional folds of the human and parasite enzymes. However, the effectiveness of compound D9 in inhibiting HAD5 compared to human PMMs (Fig. 5*A*) suggests that the inhibitor binding sites have differences that may account for this. Compounds D1 to D11 were synthesized as substrate mimetics that inhibit enzymes at the substrate binding pocket, which is supported by cocrystal structures with structurally related inhibitors bound to the active site of related proteins (74). We therefore hypothesized that D9 inhibits HAD5 at the active site. Although many active site features between the *Plasmodium* and human enzymes are conserved, an active site loop contains significant sequence variations (Fig. S9*B*). This loop in HAD5 contains Met173, Gln176, and Ile179, whereas the corresponding residues in PMM1 are Arg183, Met186, and Phe189 (Fig. S9*B*). To develop a model for how compound D9 may interact with HAD5, we computationally docked the ligand into the active site of the HAD5 X-ray crystal structure. The resulting position of D9 in the HAD5 active site is similar to the crystallographically determined binding mode of mannose-1-phosphate in the X-ray structure of human PMM1 (76). The docking solution orients the hydroxyaminomethyl group of compound D9 in proximity to the sidechains of residues in the variable active site loop (Fig. S9*B*). Furthermore, comparison of D9 to similar but less potent compounds used for HAD5 inhibitor screening, D7 and D11, indicates that the hydroxyaminomethyl group is a key determinant of HAD5 inhibition compared to the human PMM (Fig. S7). Although the combination of low resolution of the HAD5 crystal structure and computational docking precludes more detailed analysis, the resulting model suggests that interactions between the hydroxyaminomethyl group of D9 and the variable active site loop likely account for HAD5 selectivity; however, additional studies are needed to further validate this model.

**Table 2:** Summary of crystallographic statistics

Crystal	PfHAD5
Space group	P6_5_22
Cell dimensions	*a* = *b* = 161.3. Å, *c* = 109.7 Å
Data collection	
Wavelength	0.979 Å
Resolution range (highest shell)	40.3–3.50 Å (3.56–3.520 Å)
Reflections (total/unique)	354,596/11,030
Completeness (highest shell)	99.9% (100%)
<I/σ> (highest shell)	18.5 (2.7)
R_sym_ (highest shell)	33.4% (143%)
Refinement	
R_crys_/R_free_	25.4%/31.0%
No. of protein atoms	3941
No. of ligand atoms	2
R.m.s. deviation, bond lengths	0.003 Å
R.m.s. deviation, bond angles	0.74°
Avg. B-factor: protein, ligand	92.7, 59.1 Å^2^
Stereochemistry: favored, allowed, outliers	94.7, 4.5, 0.8%

## Discussion

We report here the lethal knockdown and biochemical characterization of the PMM in *P. falciparum*, HAD5. Loss of HAD5 leads to growth arrest in asexually replicating parasites, marked by defects in egress and reinvasion. This growth defect can be rescued by media supplementation with D-mannose, indicating that disruption of mannose metabolism is the primary mechanism of death in HAD5^KD^ parasites; however, a physiologically relevant concentration of 50 μM D-mannose is unable to significantly rescue growth, bolstering the case for this pathway as a therapeutic target. We further report the specific inhibition of HAD5 enzymatic activity compared to orthologous human PMMs by the hexose–phosphate analog, compound D9, highlighting the potential for specific therapeutics to be developed to HAD5.

While difficult to compare to other published PMM activities, the k_cat_ of HAD5 for its mannose–phosphate substrate is low. This may be a result of the reaction conditions, as we were implementing a linked enzyme reaction that assayed the conversion of M6P to M1P, rather than the more commonly assayed inverse reaction. We also performed these assays at room temperature, rather than 37 °C. Despite those caveats, it remains possible that HAD5 is capable of catalyzing more robust reactions with other phosphosugar substrates or bisphosphate activators, particularly because we and others have observed that HAD proteins demonstrate considerable substrate promiscuity (77, 78). However, the observation that mannose supplementation rescues HAD5^KD^ parasite growth supports our hypothesis that the essential activity of HAD5 is mediated through its impacts on mannose metabolism.

Unexpectedly, despite the dramatic decrease in HAD5 protein levels and the compelling loss of growth upon HAD5 knockdown, GPI anchor synthesis and MSP1 anchoring to merozoite surfaces are only modestly impacted. It may be that residual HAD5 activity, below the limit of detection by immunoblot analysis, is present after knockdown and sufficient for generating detectable GPI precursors and subsequent tethering of some GPI-APs. Alternatively, *P. falciparum* expresses two annotated PGMs (PF3D7_1012500 and PF3D7_0413500) (79) that could potentially catalyze PMM activity as well, which would suggest some functional redundancy to HAD5. However, the related apicomplexan organism *Toxoplasma gondii* also harbors two PGM isozymes, which lack PMM activity (80, 81), suggesting that *P. falciparum* PGMs also may lack this activity. Residual HAD5 or functional redundancy by PGMs could also explain our observation that D-mannose rescues HAD5 knockdown. Intracellular mannose is likely converted to M6P by hexokinase and thus some PMM activity would still be required for successful rescue of the GPI anchor pathway. Regardless of the source of residual GPI anchoring, the absence of another annotated PMM in the genome as well as *in vitro* biochemical activity and HAD5^KD^ growth rescue by mannose provide a compelling argument that HAD5 is a functional PMM of *P. falciparum*. While residual PMM activity present in HAD5^KD^ parasites may prevent GPI synthesis from being completely eliminated, it is insufficient to sustain parasite growth. This speaks to an exquisite sensitivity of malaria parasite cells to disruption in this pathway, whereby minor perturbations in GPI anchor synthesis nonetheless completely interrupt parasite growth, highlighting the promise of this pathway as a therapeutic target.

In addition, we found that HAD5^KD^ parasites have a complete cell cycle arrest, although others have found that untethering MSP1 from the membrane by removing its GPI-anchoring C terminus still allows for minimal parasite growth (18). We expect that this discrepancy is because of the role of HAD5 in function of all GPI-anchored parasite proteins, not solely MSP1. These other GPI-APs include related MSPs, rhoptry-associated membrane antigen, and 6-cysteine proteins, many of which are refractory to deletion and likely essential (15, 20, 34, 82). With the incomplete loss of GPI synthesis, it may be that each one of these GPI-APs, including MSP1, are only relatively de-anchored, but the modest reduction in this posttranslational modification across multiple cellular proteins works in concert to cause parasite growth arrest.

We therefore propose that HAD5, as an upstream member of the GPI biosynthesis, has great potential as an antimalarial target. We expect that HAD5 inhibition will have broad downstream effects on parasite biology across several life cycle stages. That compound D9 has markedly improved potency against malaria HAD5 compared to orthologous human enzymes provides key proof-of-concept for ongoing development of specific HAD5-directed antimalarial therapeutics. While compound D9 has limited antimalarial efficacy, this is likely because of its charged phosphonate, expected to have poor cellular penetration. This liability may be improved through a variety of medicinal chemistry strategies, including the addition of prodrug moieties to mask this charge, a strategy that has been highly effective for other phosphonate antimalarials in development (83, 84, 85). Finally, the crystal structure of HAD5 is likely to be valuable to ongoing efforts to develop more potent and specific HAD5 inhibitors as antimalarials.

This study also adds to the growing literature on HAD-like proteins in *P. falciparum*. Three related HAD proteins each independently modulate parasite sensitivity to the isoprenoid biosynthesis inhibitor FSM, which prompts the question of whether this effect would be similarly seen with other HAD proteins (68, 69, 70). Alternatively, the nonmevalonate isoprenoid biosynthesis pathway may be particularly sensitive to cellular metabolic perturbations. We found that HAD5 serves as an interesting counterexample. HAD5 knockdown yields no changes to FSM sensitivity, providing evidence of a HAD protein and a metabolic perturbation that does not impact the sensitivity of parasites to inhibition of isoprenoid metabolism.

Finally, we note that HAD5 and the GPI anchor biosynthesis pathway are expressed throughout the parasite’s life cycle. Several GPI-APs are expressed in gamete-stage and oocyst-stage parasites (86, 87), and circumsporozoite protein, the target of the RTS,S malaria vaccine and the more recent R21 vaccine candidate (25, 26, 27, 28), is a GPI-AP expressed on the surface of sporozoites that facilitates sporozoite development (29) and targets sporozoites to the liver (88). Furthermore, sexual-stage parasites harbor additional fates of M1P, including *C*-mannosylation (89, 90) and *O*-fucosylation (91, 92), suggesting that HAD5 will play a critical role in parasite biology across several life stages. Hence, HAD5 serves an essential role in parasite metabolism and shows promise as a specific therapeutic target. The inhibition of HAD5 not only has potential to treat intraerythrocytic *P. falciparum* infection but also may serve to block transmission.

## Experimental procedures

### Parasite strains and culturing

Unless otherwise indicated, parasites were maintained at 37 °C in 5% O_2_, 5% CO_2_, 90% N_2_ in a 2% suspension of human RBCs in RPMI medium (Gibco) modified with the addition of 30 mM NaHCO_3_, 11 mM glucose, 5 mM Hepes, 1 mM sodium pyruvate, 110 μM hypoxanthine, 10 μg/ml gentamicin, (Sigma), and 2.5 g/l AlbuMAX I (Gibco). Deidentified RBCs were obtained from the Barnes-Jewish Hospital blood bank, St Louis Children’s Hospital blood bank, and American Red Cross Blood Services.

The HAD5 conditional knockdown strain, “HAD5^KD^,” was generated by transfecting NF54^attB^ parasites (93) using methods as described (46, 47). Resultant parasites were maintained in the presence of aTc (Cayman Chemicals) in dimethylsulfoxide at 500 nM unless otherwise specified. Parasites were synchronized with a combination of 5% sorbitol (Fisher Bioreagents) and 1.5 μM Compound 1 (MedChemExpress) (94, 95).

### Plasmodium growth measurement

Parasitemia in daily growth assays was measured *via* flow cytometry by incubating 10 μl of parasite culture with 190 μl of 0.4 μg/ml acridine orange (Invitrogen) in PBS for 1 min. Stained parasites were analyzed on a BD FACS Canto flow cytometer gating on DNA- and RNA-bound dye signal using FITC and PerCP-Cy5.5 filters, respectively. Fifty thousand events were recorded for each sample.

### Merozoite reinvasion

Merozoite reinvasion was assessed as described (96). Parasites were synchronized, grown in ±aTc, and treated with 10 μM epoxysuccinyl-L-leucylamido(4-guanidino)butane (E64) (Sigma) for 8 h to stall them in late schizont stages. E64 was washed out, and cultures were passed through a 1.2 μm filter to lyse the schizonts. Lysates were incubated with fresh RBCs for 1 h to allow reinvasion before cultures were washed again to remove debris. Parasitemia was assessed 24 h after reinvasion by acridine orange staining and flow cytometry. Reinvasion efficiency was assessed by normalizing resultant parasitemia to the measured parasitemia prior to lysis.

### Light and fluorescent microscopy

Parasite development was monitored by thin smear of synchronized parasites that were dyed with modified Giemsa Stain (Sigma). For fluorescent microscopy, E64-treated schizont-stage parasites were mechanically lysed by passing them through a 1.2 μm filter (96). Lysates were added to poly-lysine–coated glass slides, fixed in 4% paraformaldehyde, 0.0075%glutaraldehyde in PBS, and permeabilized in 0.1% Triton X-100. Immunofluorescence was performed using mouse anti-MSP1 monoclonal antibody (Novus Biologicals) and goat-anti-mouse Alexafluor 488 secondary antibody (Life Technologies). Samples were then preserved with ProLong Gold antifade reagent with DAPI (Life Technologies).

All immunofluorescence and bright field images were taken using a Zeiss Axio Observer D1 inverted microscope (Carl Zeiss Inc), equipped with a Axiocam 503 color camera, at the Washington University Molecular Microbiology Imaging facility. Images were acquired with a Plan-Apochromat 100× (NA 1.4) objective using the ZEN 2.3 pro (blue edition) software. Fluorescent signal was quantified using the ImageStudioLite software from LI-COR.

### Transmission electron microscopy

For ultrastructural analyses, highly synchronized infected RBCs were fixed in 2% paraformaldehyde/2.5% glutaraldehyde (Polysciences Inc) in 100 mM sodium cacodylate buffer, pH 7.2 for 1 h at room temperature. Samples were washed in sodium cacodylate buffer and postfixed in 1% osmium tetroxide (Polysciences Inc) for 1 h at room temperature. Samples were then rinsed in dH_2_O, dehydrated in a graded series of ethanol, and embedded in Eponate 12 resin (Ted Pella Inc). Sections of 95 nm were cut with a Leica Ultracut UCT ultramicrotome (Leica Microsystems Inc), stained with uranyl acetate and lead citrate, and viewed on a JEOL 1200 EX transmission electron microscope (JEOL USA Inc) equipped with an AMT eight megapixel digital camera and AMT Image Capture Engine V602 software (Advanced Microscopy Techniques).

### Cloning

The coding sequence of HAD5 was cloned from cDNA of 3D7 parasites using primers P1 and P2 (Table S1) and cloned into the BG1861 vector (97), which introduces an N-terminal 6xHis-tag, by ligation-independent cloning. This coding sequence was subsequently cut and pasted into a pET28a vector with NdeI and BamHI-HF (NEB), followed by ligation with NEB Quick Ligase using manufacturer’s protocols. The coding sequence did not match published reference sequence of PF3D7_1017400, as an adenosine-to-guanosine mutation yielded an Asn-to-Ser substitution at residue 100. To revert this sequence to the reference sequence, primer P3 (Table S1) was used in the QuikChange Multisite-directed mutagenesis kit (Agilent), and the resulting plasmid was transformed into XL10 Gold ultracompetent *Escherichia coli* cells. The HAD5^D11A^ allele was generated from the WT plasmid, again using the QuikChange multisite-directed mutagenesis kit and primer P4 (Table S1). *Homo sapiens* PMM1 and PMM2 coding sequences were identified from UniProt, codon optimized for *E. coli*, and synthesized by Integrated DNA Technologies (Table S2). These gene blocks were amplified and extended using primers P5 + P6 (PMM1) and P7 + P6 (PMM2) and PrimeSTAR GXL DNA polymerase (Takara) (Table S1). These sequences were then cloned into the pET28a plasmid by Gibson assembly at NdeI and BamHI-HF cut sites, and plasmids were transformed into XL10 Gold ultracompetent *E. coli* cells.

*E. coli* mannose-1-phosphate guanylyl transferase (ManC) was cloned for use in PMM assays. The coding sequence of *Ec*ManC was identified from UniProt, and a gene block of the sequence was ordered from Integrated DNA Technologies (Table S2). This sequence was cloned by ligation-independent cloning into a BG1861 vector that has been modified with a starting KFS sequence downstream of the 6xHis-tag to enhance protein expression (98), and the resulting plasmid was transformed into Stellar Competent Cells (Takara).

### Recombinant protein expression and purification

To generate recombinant protein for enzyme assays and crystallography, the pET28a plasmids were transformed into BL21 Gold (DE3) competent *E. coli* cells (Agilent). Cells were grown to absorbance at 600 nm (*A*_600_) of 0.6 to 0.8 in Terrific Broth medium (24 g/l yeast extract, 20 g/l tryptone, 4 ml/l glycerol, 17 mM KH_2_PO_4_, 72 mM K_2_HPO_4_) shaking at 37 °C and then induced for approximately 18 h with 1 mM IPTG at 16 °C. Cells were collected by centrifugation and resuspended in lysis buffer containing 25 mM Tris–HCl (pH 7.5), 250 mM NaCl, 1 mM MgCl_2_, 10% glycerol, 20 mM imidazole, and 100 μM PMSF and lysed by sonication. Lysate was centrifuged at 18,000*g* for 1 h, and the supernatant containing soluble protein was bound to nickel agarose beads (Gold Biotechnology), washed with buffer containing 25 mM Tris–HCl (pH 7.5), 250 mM NaCl, 1 mM MgCl_2_, 10% glycerol, and 20 mM imidazole, and eluted with 10 ml of buffer containing 25 mM Tris–HCl (pH 7.5), 250 mM NaCl, 1 mM MgCl_2_, 10% glycerol, and 320 mM imidazole. Eluate was dialyzed overnight at 4 °C in the presence of 30 units of thrombin protease (Sigma) into buffer containing 25 mM Tris–HCl (pH 7.5), 250 mM NaCl, 1 mM MgCl_2_, and 10% glycerol. The dialyzed, thrombin-cleaved mixture was then run over nickel agarose beads and benzamidine sepharose beads (GE Healthcare) to remove the cleaved His-tag and thrombin. The flow through protein was collected and further purified by size-exclusion chromatography using a HiLoad 16/60 Superdex-200 column (GE Healthcare), equilibrated in dialysis buffer. Elution fractions containing the protein of interest were identified by UV absorbance and SDS-PAGE, pooled, and concentrated to 6 mg/ml. Protein solutions were flash frozen in liquid nitrogen and stored at −80 °C (Figs. 1*B* and S7*A*).

*Ec*ManC for enzyme activity assays was expressed by cloning the BG1861:ManC vector into BL21(DE3) pLysS *E. coli* cells (Life Technologies). Cells were grown in LB broth to an *A*_600_ of 0.7 to 0.8 and induced with 1 mM IPTG for 2 h at 37 °C. Cells were harvested, and protein was purified as described for other proteins above. However, as this construct lacked the thrombin-cleavable site from the pET28a vector, the elution from nickel beads was directly run over the size exclusion column, then pooled and concentrated (Fig. S7*B*).

### HAD5 activity assays

PMM activity of HAD5 was measured using a linked enzyme scheme (Fig. S1*A*), modified from the EnzChek phosphate release kit (ThermoFisher Scientific). Two hundred micromolar of 2-amino-6-mercapto-7-methyl-purine riboside and 1× EnzChek reaction buffer (50 mM Tris HCl, 1 mM MgCl_2_, pH 7.5, containing 100 μM sodium azide) were incubated with 1 U/ml purine nucleoside phosphorylase, 1 U/ml pyrophosphatase, 125 μM GTP, 52 μg/ml recombinant purified *E. coli* ManC, and 1 mM mannose-6-phosphate. 12.5 μM G-1,6-P was also added to demonstrate its activating properties on HAD5. All reagents were obtained from Sigma-Aldrich. The reaction was started by adding 50 ng recombinant HAD5 (WT or D11A), which is within the linear range of the assay (Fig. S1*B*). Reactions took place in 40 μl, and the production of nucleotide by purine nucleoside phosphorylase was measured at 360 nM.

PGM assays (Fig. S1*C*) were developed by incubating 1 mM glucose 1-phosphate, 0.75 mM NADP^+^, 2.5 U/ml glucose-6-phosphate dehydrogenase, and 10 μM G-1,6-P in 50 μl reactions containing 25 mM NaCl, 25 mM Tris–HCl, pH 7.5, and 1 mM MgCl_2_. All reagents were obtained from Sigma-Aldrich. Again, 50 ng of HAD5 was added to start the reaction (Fig. S1*D*), and the production of reduced NADPH was measured by absorbance at 340 nm.

All reactions took place at room temperature in clear CoStar 96-well half-area plates, and absorbances were measured by a PerkinElmer multimode Envision plate reader. For Michaelis-Menten kinetics of each assay, 2-fold serial dilutions of substrate concentrations (either M6P or glucose 1-phosphate, respectively) were made, and all other components of the assays were kept constant.

### Inhibition assays of recombinant HAD5

Synthesis of compounds D1 to D11 (Fig. S8*A*) has been described previously (73, 74), with the exception of compound D9, whose synthesis is described, and characterization data are included, in the attached supporting information. To assess inhibition of HAD5, compounds D1 to D11 were suspended in water and added to the PGM assay of HAD5 activity (the PMM assay was not used, as cross-inhibition was seen with downstream components of that assay but not with the PGM assay; Fig. S8*D*). Five microliters of water volume in the assay was replaced with 5 μl serial dilutions of analogs, with final concentrations ranging from 0 μM to 1 mM. The rate of product formation in each condition was used to determine the IC_50_ for each inhibitor. In addition to HAD5, these assays were performed with recombinantly purified human PMM1 and PMM2. For these inhibition assays, 200 ng of HAD5 (139 nM), PMM1 (133 nM), or PMM2 (65 nM) was used to start the reaction.

Time-dependent inhibition was assessed by preincubating HAD5 or PMM1 in 416 μM D9 for time points up to 60 min, before adding the enzyme + inhibitor mixture to the full reaction mixture, achieving a final [D9] of 50 μM.

### Parasite growth inhibition assays

Dose–response inhibition experiments were performed on asynchronous parasites. Three experimental replicates were performed, with technical duplicates, for each experiment.

Both GlcN and ManN inhibition experiments were performed by adding 2-fold dilutions of each compound in water, from 0 mM to 2 mM, to a 100 μl culture of parasites. One micromolar of chloroquine was used as a positive control. Inhibition of cultured parasites by compound D9 was assessed using 100 μM D9 or equivalent volume of water. The parasite growth in these experiments was assessed by acridine orange staining and flow cytometry measurement of parasitemia 72 h after treatment.

Dose–response of FSM was assessed using concentrations up to 50 μM FSM in water, and the growth was measured after 72 h treatment on a PerkinElmer multimode Envision plate reader by Quant-iT PicoGreen dsDNA reagent (ThermoFisher Scientific) staining.

### Western blotting

To verify tagging and knockdown of protein, cultures of HAD5^KD^ were grown for 24 h in ±aTc conditions, then RBCs were lysed with cold PBS + 0.1% saponin. Samples were centrifuged to pellet parasites and remove excess hemoglobin, then the parasites were lysed in RIPA (50 mM Tris, pH 7.4; 150 mM NaCl; 0.1% SDS; 1% Triton X-100; 0.5% DOC) plus HALT-Protease Inhibitor Cocktail, EDTA-free (ThermoFisher Scientific). Lysates were centrifuged at high speed to pellet and remove hemozoin. Cleared lysates were then diluted in SDS sample buffer (10% SDS, 0.5 M DTT, 2.5 mg/ml bromophenol blue, 30% 1 M Tris pH 6.8, 50% glycerol) and boiled for 5 min. Lysates were separated by SDS-PAGE, then transferred to 0.45 μm nitrocellulose membrane (ThermoFisher Scientific). The membranes were blocked in PBS + 3% bovine serum albumin, then probed with primary antibodies: rabbit anti-HA (Sigma), mouse anti-plasmepsin V (99, 100). Membranes were washed in PBS + 0.5% Tween 20, then probed with secondary antibodies goat anti-rabbit IRDye 800CW 1:20,000 (LI-COR) and donkey anti-mouse IRDye 680LT 1:20,000 (LI-COR). Membranes were then washed in PBS + 0.1% Tween 20 and imaged on a LI-COR Odyssey platform.

### Autoradiography and GPI anchor quantification

GPI anchor autoradiography was performed as previously described (17, 57, 59). Briefly, 33- to 39-h-old parasites that had been grown in ±aTc conditions were washed and resuspended in glucose-free media (RPMI R1383 + 20 mM D-fructose, 25 mM Hepes, 21 mM NaHCO_3_, 0.37 mM hypoxanthine, 11 mM glutathione, 5 g/l Albumax I, and 10 μg/ml gentamicin). One hundred fifty microcurie of 40 Ci/mmol [^3^H]GlcN (American Radiochemicals) was added to each 10 ml culture and incubated for 3 h. Parasites were saponin lysed, and glycolipids were extracted with chloroform:methanol:water (10:10:3), nitrogen evaporation, and n-butanol partitioning (60). The resultant glycolipids were run on TLC Silica gel 60 F_254_ plates (Millipore Sigma) using chloroform:methanol:water (10:10:3) as a solvent. The TLC plates were exposed to autoradiography films (MidSci), which were developed after 1 week of exposure. Films were imaged on a BIO-RAD ChemiDoc MP imaging system, and the signal was quantified by the ImageLab software from Bio-Rad.

### TritonX-114 membrane partitioning

Membrane partitioning was adapted from Doering *et al.* (64). Briefly, at approximately 40 to 44 h after invasion, synchronized parasite cultures were magnetically purified (Miltenyi Biotec), and the elution was centrifuged and resuspended in 1 ml ice-cold Tris-buffered saline (TBS) + 1× HALT protease inhibitor cocktail, EDTA-free (ThermoFisher Scientific). Two hundred microliter precondensed Triton X-114 was added to lyse parasites, and lysates were incubated on ice for 15 min. Lysates were centrifuged to remove debris. Then followed a series of five extractions with cold TBS + protease inhibitor and precondensed TritonX-114, followed by warming to 37 °C and centrifugation to separate phases. The lysate, detergent-enriched phase and the soluble phase were analyzed by SDS-PAGE and Western blotted as described previously using mouse anti-plasmepsin V 1:20 (99) and rabbit anti-HAD1 1:10 (68) or rabbit anti-MSP1 1:1000 MSP1 (101). Detergent samples were diluted 5-fold in TBS prior to SDS-PAGE to avoid TritonX-114 interference with the gel.

### Protein crystallography and ligand docking

Crystals of *P. falciparum* HAD5 were grown at 4 °C using vapor diffusion in hanging drops comprised of 2 μl protein (6 mg/ml) and 2 μl crystallization buffer (0.1 M Bis–Tris propane pH 6.5, 0.1 M calcium acetate, 16% (w/v) PEG 8000, and 2% (v/v) benzamidine hydrochloride). Prior to data collection, crystals were flash frozen in mother liquor supplemented with 25% glycerol. All diffraction images were collected at 100K at beamline 19-ID of the Structural Biology Center at Argonne National Laboratory Advanced Photon Source. HKL3000 was used to index, integrate, and scale the datasets (102). Prior to phasing, a homology model of PfHAD5 was created using SWISS-MODEL (103) based on human alpha PMM 1 (PDB: 6CFR (104), sequence identity 51%). Molecular replacement was performed using PHASER (105) and the homology model of PfHAD5 as a search model. COOT and PHENIX were used for iterative rounds of model building and refinement (106, 107). Data collection and refinement statistics are summarized in Table 2. Atomic coordinates and structure factors of PfHAD5 have been deposited in the RCSB Protein Data Bank (PDB: 7MYV).

The PfHAD5 structure was prepared for docking using AutoDock Tools 1.5.7 (108). The three-dimensional structure of compound D9 was prepared using Avogadro (109) and was prepared for docking using AutoDock Tools 1.5.7. Docking was performed with AutoDock Vina using default search settings (110).

## Data availability

Atomic coordinates and structure factors of PfHAD5 have been deposited in the RCSB Protein Data Bank (PDB: 7MYV). All other data have been provided in figures and supporting information.

## Supporting information

This article contains supporting information (111, 112, 113, 114).

## Conflict of interest

A. R. O. J. reports financial support was provided by 10.13039/100000002National Institutes of Health and 10.13039/100000861Burroughs Wellcome Fund. D. L. J. reports financial support was provided by 10.13039/501100000038Natural Sciences and Engineering Research Council of Canada and 10.13039/501100000024Canadian Institutes of Health Research. A. R. O. J. reports a relationship with Pluton Biosciences that includes board membership and with American Society for Microbiology that includes consulting or advisory. A. R. O. J. has patent issued and pending patents on antimalarials pending to none.

## Supporting information

Supplemental Figures S1–S26, Scheme S1, Tables S1 and S2
