# Supplementary material for: Enzymatic and structural characterization of HAD5, an essential phosphomannomutase of malaria-causing parasites
Source: J Biol Chem. 2021 Dec 29;298(2):101550. doi: 10.1016/j.jbc.2021.101550 (PMC8808168; doi:10.1016/j.jbc.2021.101550)
Supplement: Supplemental Figures S1–S26, Scheme S1, Tables S1 and S2 [file mmc1.docx]

# SUPPORTING INFORMATION

**Enzymatic and structural characterization of HAD5, an essential phosphomannomutase of malaria-causing parasites**

Philip M Frasse^1^, Justin J Miller^2^, Alexander J Polino^1^, Ebrahim Soleimani^3,4^, Jian-She Zhu^3^, David L Jakeman^3,5^, Joseph M Jez^2^, Daniel E Goldberg^1^, Audrey R Odom John^6a^

^1^Division of Infectious Diseases, Departments of Medicine and Molecular Microbiology, Washington University School of Medicine, St. Louis, Missouri, USA.

^2^Department of Biology, Washington University in St. Louis, St. Louis, MO 63130, USA.

^3^College of Pharmacy, Dalhousie University, Halifax, Nova Scotia, Canada

^4^Department of Chemistry, Razi University, Kermanshah, Iran

^5^Department of Chemistry, Dalhousie University, Halifax, Nova Scotia, Canada

^6^Division of Infectious Diseases, Department of Pediatrics, Children’s Hospital of Philadelphia, University of Pennsylvania, Philadelphia, Pennsylvania, USA

^a^Correspondence and requests for materials should be addressed to AROJ

**List of Materials**

Table S1: Primers used for cloning.

Table S2: gBlock sequences used in cloning.

Figure S1: Enzymatic activity assays​.

Figure S2: Chemical rescue of parasite growth.​

Figure S3: Ultrastructures of HAD5^KD^ schizonts by transmission electron micrograph.

Figure S4: HAD5 KD Parasites are deficient in reinvasion​.

Figure S5: TritonX-114 partition of MSP1​.

Figure S6: Knockdown of HAD5 has no effect on FSM sensitivity​.

Figure S7: Purified recombinant proteins​.

Figure S8: Structures and evaluation of compounds D1-D11​.

Figure S9: Compound D9 does not inhibit parasites in culture​.

Synthesis of phosphonate analogue of 6NHOH-G1CP (D9)

Scheme 1. Synthesis of phosphonate analogue of 6NHOH-G1CP (D9)

Figure S10: ^1^H NMR of compound **2**

Figure S11: ^31^P NMR of compound **2**

Figure S12: ^13^C NMR of compound **2**

Figure S13: ^1^H NMR of compound **3**

Figure S14: ^31^P NMR of compound **3**

Figure S15: ^13^C NMR of compound **3**

Figure S16: ^1^H NMR of compound **4**

Figure S17: ^31^P NMR of compound **4**

Figure S18: ^1^H NMR of compound **5**

Figure S19: ^31^P NMR of compound **5**

Figure S20: ^13^C NMR of compound **5**

Figure S21: ^1^H NMR of compound **6**

Figure S22: ^31^P NMR of compound **6**

Figure S23: ^13^C NMR of compound **6**

Figure S24: ^1^H NMR of compound **D9**

Figure S25: ^31^P NMR of compound **D9**

Figure S26: ^13^C NMR of compound **D9**

**Table S1**: **Primers used for cloning**

| **Primer Name** | **Sequence** | **Notes** |
| --- | --- | --- |
| P1 | CTCACCACCACCACCACCATATGAATAAGAAAAAAGGCAATATTTCTGT | For cloning HAD5 sequence from *P. falciparum* cDNA into BG1861 vector |
| P2 | ATCCTATCTTACTCACTTACAAGAAATTCTCTCTTAAAATTTTAAC | For cloning HAD5 sequence from *P. falciparum* cDNA into BG1861 vector |
| P3 | GAGAAGACCGATTAAAAAAATTGATAAATTATAGTTTAAAATATATTGCC | For reverting the HAD5 gene sequence to the reference sequence. |
| P4 | GGCAATATTTCTGTTTGCTGTAGATGGGACCC | For generating HAD5^D11A^ mutant for recombinant protein. |
| P5 | GCCGCGCGGCAGCCATATGGCAGTTACA | Forward primer for cloning HsPMM1 gblock into pET28a vector. |
| P6 | CGGAGCTCGAATTCGGATCCTATCTTACTC | Reverse primer for cloning HsPMM1 and Hs PMM2 gblocks into pET28a vector. |
| P7 | CGGAGCTCGAATTCGGATCCTATCTTACTCACTTA | Forward primer for cloning HsPMM2 gblock into pET28a vector. |

**Table S2: gBlock sequences used in cloning**

| **Gene Name** | **Sequence** |
| --- | --- |
| *Hs*PMM1 | ATGGCAGTTACAGCCCAGGCAGCCCGTCGTAAGGAGCGTGTCTTATGTCTGTTCGATGTAGACGGAACTCTGACCCCCGCACGTCAAAAAATCGACCCGGAAGTTGCAGCTTTTTTGCAGAAGCTGCGTTCGCGCGTCCAGATCGGTGTAGTCGGCGGATCAGATTACTGCAAAATCGCCGAGCAACTTGGAGATGGCGACGAAGTGATCGAGAAGTTTGACTACGTCTTCGCCGAGAATGGGACAGTTCAATACAAGCATGGGCGCTTATTGAGTAAGCAGACTATTCAGAACCATCTGGGGGAGGAGTTGCTTCAAGATCTTATTAATTTTTGTTTATCCTATATGGCCTTACTTCGCCTGCCCAAAAAGCGCGGTACTTTCATTGAGTTCCGTAACGGGATGCTGAACATCAGTCCAATCGGTCGCTCATGCACTCTGGAGGAGCGTATCGAGTTTTCTGAACTTGACAAGAAAGAGAAAATTCGTGAGAAATTCGTCGAGGCGTTAAAAACGGAGTTTGCAGGGAAGGGATTACGCTTTTCTCGCGGAGGCATGATTTCATTCGACGTGTTTCCAGAAGGTTGGGACAAGCGCTACTGCTTGGACTCATTAGATCAAGATAGCTTTGATACCATTCACTTTTTCGGGAACGAAACCTCGCCTGGGGGTAACGACTTCGAGATCTTTGCGGACCCTCGTACGGTCGGGCACTCGGTAGTGAGCCCTCAGGACACCGTGCAACGTTGTCGTGAGATTTTTTTCCCAGAGACGGCGCATGAAGCGTAAGTGAGTAAGATAGGATCCGAATTCGAGCTCCG |
| *Hs*PMM2 | ATGGCGGCTCCGGGCCCAGCATTATGTTTATTTGACGTTGACGGAACCCTTACCGCACCGCGTCAAAAGATCACGAAGGAAATGGATGATTTTTTGCAGAAGTTACGTCAGAAGATCAAAATCGGGGTGGTCGGTGGTTCCGATTTTGAGAAAGTTCAGGAGCAGCTTGGAAACGACGTGGTTGAGAAGTACGATTACGTCTTTCCGGAAAATGGGTTGGTCGCGTATAAGGACGGTAAACTGCTTTGTCGTCAAAATATTCAGTCCCATCTGGGCGAAGCCTTGATTCAAGATTTAATCAATTATTGCTTATCCTATATCGCTAAGATCAAATTGCCCAAGAAACGCGGCACCTTTATTGAGTTTCGTAATGGCATGTTGAACGTGTCCCCGATCGGACGTTCGTGTTCCCAGGAGGAACGCATTGAGTTCTATGAACTGGATAAAAAAGAAAATATCCGTCAAAAGTTCGTTGCCGATCTTCGCAAGGAGTTCGCAGGCAAAGGTTTAACGTTCTCAATCGGCGGTCAAATCTCTTTCGATGTGTTCCCAGACGGATGGGACAAACGTTACTGTCTTCGCCATGTAGAAAATGATGGATATAAAACCATCTACTTTTTTGGGGACAAAACAATGCCAGGAGGGAATGACCATGAAATTTTCACGGACCCCCGTACAATGGGCTACTCAGTAACCGCACCGGAAGATACCCGTCGTATTTGCGAGCTGTTGTTCTCTTAAGTGAGTAAG |
| *Ec*ManC | CTCACCACCACCACCACCATATGGCTCAAAGTAAACTTTACCCAGTGGTAATGGCGGGGGGAAGTGGCTCTCGTCTTTGGCCTTTATCTCGCGTTCTTTATCCAAAACAGTTCTTGTGCCTTAAGGGTGATTTGACAATGTTGCAAACAACGATCTGCCGCCTGAATGGTGTCGAATGTGAGTCACCTGTGGTAATTTGCAATGAACAGCACCGTTTCATCGTAGCAGAACAACTGCGTCAGCTGAATAAATTAACGGAAAATATTATCCTGGAGCCTGCTGGTCGCAACACGGCACCTGCAATCGCCTTGGCTGCACTTGCGGCCAAGCGTCACTCACCAGAAAGTGACCCGCTTATGCTTGTCTTGGCCGCCGATCATGTGATCGCAGATGAAGATGCTTTTCGTGCTGCTGTCCGTAATGCGATGCCATATGCCGAAGCTGGAAAGTTAGTTACGTTTGGTATCGTGCCGGATTTGCCGGAGACTGGATATGGTTATATCCGCCGTGGGGAGGTCAGCGCTGGAGAACAAGACATGGTAGCCTTCGAGGTGGCACAATTTGTTGAAAAGCCAAATCTTGAGACAGCACAAGCGTACGTGGCTAGTGGGGAGTATTACTGGAACTCTGGGATGTTTTTATTCCGTGCGGGGCGTTACCTTGAAGAACTGAAAAAATATCGTCCTGACATTTTAGACGCGTGTGAAAAGGCAATGAGTGCAGTGGACCCAGACTTAAACTTTATTCGTGTAGACGAAGAGGCTTTCTTGGCATGTCCAGAAGAATCCGTTGACTATGCCGTGATGGAGCGTACGGCTGATGCGGTAGTTGTCCCAATGGACGCTGGATGGTCCGATGTTGGCAGCTGGTCATCGCTTTGGGAAATTAGCGCCCACACCGCGGAAGGAAATGTATGTCACGGCGACGTGATTAACCATAAAACAGAAAATTCATACGTTTATGCGGAATCCGGCTTGGTCACTACTGTCGGAGTGAAGGATTTGGTTGTGGTGCAAACGAAAGATGCAGTATTAATTGCGGACCGCAATGCTGTCCAAGATGTGAAGAAAGTAGTTGAACAGATTAAGGCTGATGGTCGTCACGAGCATCGCGTCCACCGCGAAGTATATCGCCCATGGGGAAAGTATGACTCTATTGATGCGGGTGACCGCTATCAAGTTAAACGTATCACAGTCAAACCCGGTGAGGGGCTTTCGGTGCAAATGCATCATCATCGCGCAGAGCATTGGGTAGTGGTTGCGGGTACTGCGAAAGTAACAATTGATGGCGATATCAAGTTGCTTGGCGAAAATGAGTCAATTTACATCCCGCTGGGCGCGACACACTGTCTTGAGAATCCGGGGAAAATCCCATTGGATTTAATCGAAGTGCGTTCTGGATCTTACTTAGAGGAGGATGACGTTGTTCGTTTTGCTGATCGTTACGGACGCGTTtAAGTGAGTAAGATAGGAT |


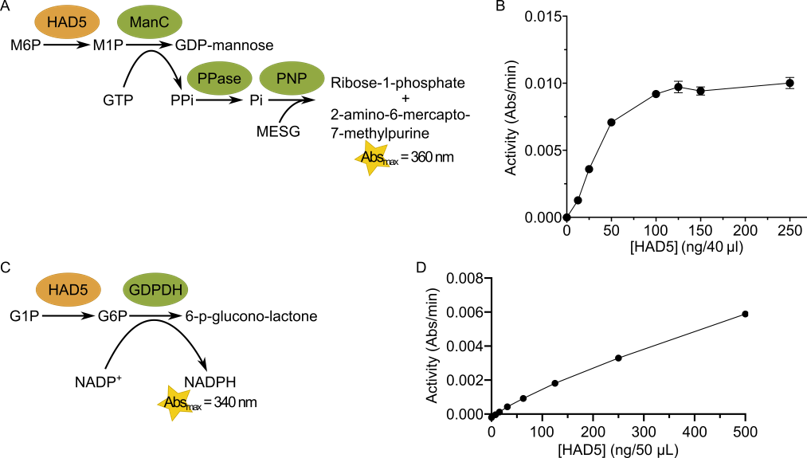


**Figure S1. Enzymatic activity assays**​. **(A)** Schematic of the phosphomannomutase (PMM) activity assay, in which HAD5 converts mannose 6-phosphate (M6P) to mannose 1-phosphate (M1P). A series of linked-enzyme steps translates that activity into a spectrophotometric signal at 360 nm. ​**(B)** Graph depicting enzymatic activity in the PMM assay with varying HAD5 concentration, demonstrating that our chosen value of 50 ng / 40 µL reaction is within the linear range of the assay with respect to enzyme concentration.​ **(C)** Schematic of the phosphoglucomutase (PGM) activity assay, in which HAD5 converts glucose 1-phosphate (G1P) to glucose 6-phosphate (G6P), which is then used by glucose 6-phosphate dehydrogenase (G6PDH) to generate NADPH, which can be measured at 340 nm. ​**(D)** Graph depicting enzymatic activity in the PGM assay with varying HAD5 concentration, demonstrating that our chosen value of 50 ng / 50 µL reaction is within the linear range of the assay with respect to enzyme concentration.


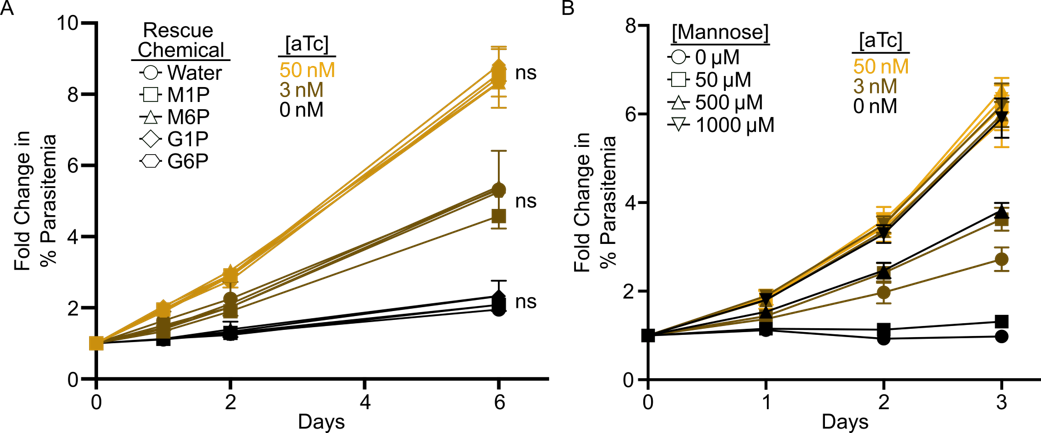


**Figure S2. Chemical rescue of parasite growth.**​ **(A)** HAD5^KD^ parasite growth was measured in the presence of varying anhydrotetracycline (aTc) concentrations with chemical rescue by four different chemicals: mannose 1-phosphate (M1P), mannose 6-phosphate (M6P), glucose 1-phosphate (G1P), and glucose 6-phosphate (G6P) at 20 µM concentration. Data depicts the mean ± standard error of the mean (SEM) of duplicate experiments and represents fold change in parasitemia over time. Statistics were performed on the Day 6 parasitemia, using a two-way ANOVA with Dunnett's multiple comparisons test, with individual variances computed for each comparison. In all cases, chemical rescues were compared to the vehicle control within a given aTc concentration, with no significant rescue of growth observed (ns = not significant). ​**(B)** Shown are the complete data of the fold change in parasitemia over time when HAD5^KD^ parasites are grown in varying aTc and D-mannose concentrations. Data represent mean ± SEM of three independent experiments with technical duplicates. The data from the Day 3 time point was used to generate Figure 2B.


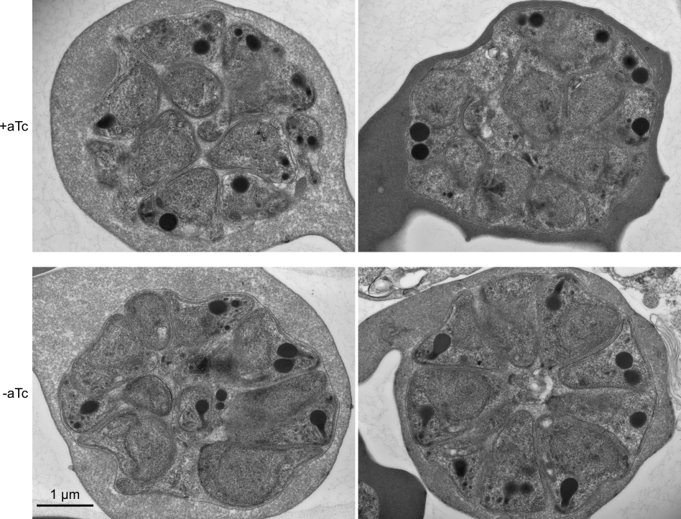


**F**i**gure S3. Ultrastructures of HAD5^KD^ schizonts by transmission electron micrograph.** Transmission electron microscopy of highly synchronized schizont parasites showing successful schizogony in parasites grown under -aTc conditions compared to +aTc.


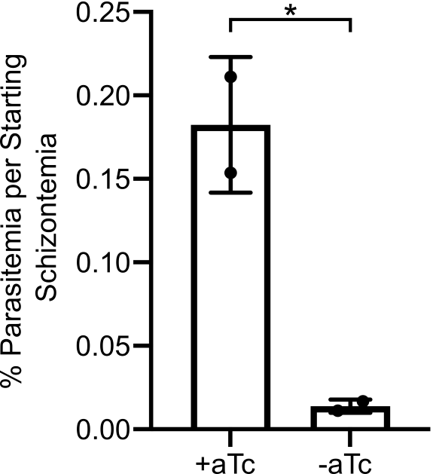


**Figure S4. HAD5 KD Parasites are deficient in reinvasion**​. Schizont-stage parasites 40-44 hours post invasion, grown in ±aTc conditions, were E64-arrested as late schizonts for 8 hours, mechanically lysed, and allowed to reinvade over fresh red blood cells. 24 hours later, parasitemia was assessed by flow cytometry and normalized to pre-lysis schizontemia, demonstrating a deficiency in reinvasion by parasites grown in –aTc conditions. Statistics were performed using an unpaired two-tailed t-test. **p*=0.028​


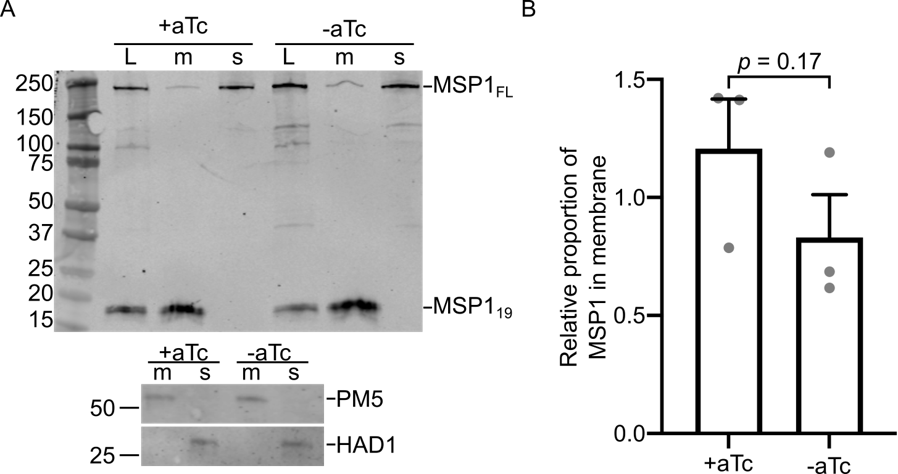


**Figure S5. TritonX-114 partition of MSP1**​. **(A)** Representative Western blot of whole lysate (L), membranous fraction (m), and soluble fraction (s) of 40-44hr parasites grown in ±aTc, and blotted for MSP1. Full Length (FL) and 19 kDa fragments of MSP1 are indicated. Plasmepsin 5 (PM5) and HAD1 were used as membrane and soluble controls, respectively.​ Molecular Weight ladder sizes are indicated in kDa on the left. **(B)** Quantification of A. Full Length and MSP1_19_ were summed in each lane, and the relative signal in membranous lanes were compared to that of whole lysate. Data represent mean + SEM of three independent experiments. Statistics were performed using a paired, two-tailed t-test.


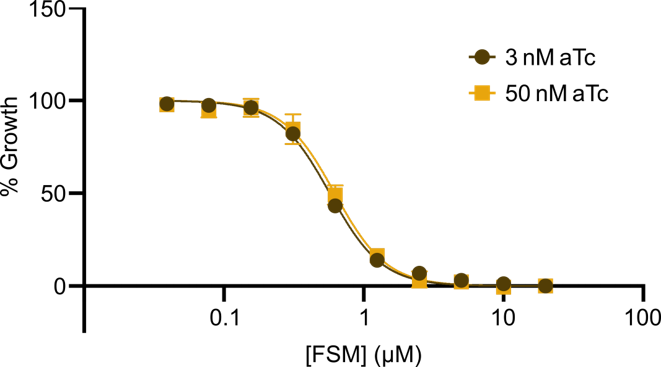


**Figure S6. Knockdown of HAD5 has no effect on FSM sensitivity**​. 72-hour dose-response curves of asynchronous parasites treated with fosmidomycin in either intermediate or saturating aTc concentrations. % Growth was relative to a vehicle control. The EC_50_ ± SEM (in µM) for each condition were: 3 nM, 0.57 ± 0.02​; 50 nM, 0.62 ± 0.04. Not significant (*p* = 0.39) by unpaired t-test.


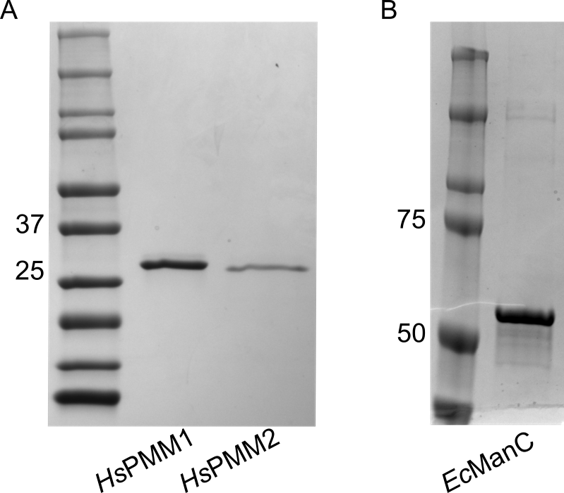


**Figure S7. Purified recombinant proteins**​. Coomassie-stained SDS-PAGE gels of final purified forms of recombinant *Hs*PMM1, *Hs*PMM2 **(A)** and *Ec*ManC **(B)**. ​


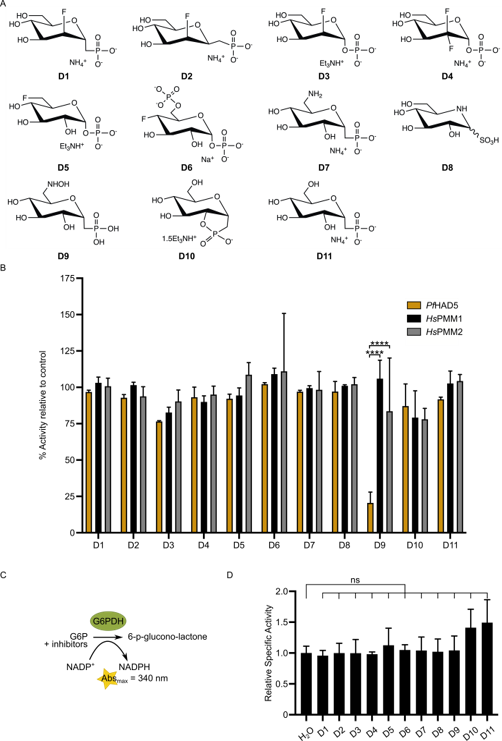


**Figure S8. Structures and evaluation of compounds D1-D11​.** **(A)** Structures of the 11 compounds tested for inhibition against *Pf*HAD5, *Hs*PMM1, and *Hs*PMM2​. **(B)** Activity of recombinant *Pf*HAD5, *Hs*PMM1, and *Hs*PMM2 when treated with 125 µM of the indicated compounds as a percentage of treatment with a vehicle control. Data represent the mean ± SEM of 3 independent experiments with technical replicates. Statistics were performed with a two-way ANOVA using Tukey's test for multiple comparisons. *****p*<0.0001. All other comparisons between enzymes for a given compound were not significant. ​**(C)** Diagram of the amended assay to assess compound inhibition of downstream components of the assay. Assays were commenced with the addition of substrate (G6P).​ **(D)** Quantification of assay shown in C. Assay activity is depicted relative to treatment with a vehicle control. Data represent the mean ± SEM of 3 independent experiments with technical replicates. Statistics were performed with an ordinary one-way ANOVA using Dunnett's test for multiple comparisons with single pooled variance. ns = not significant.​

​


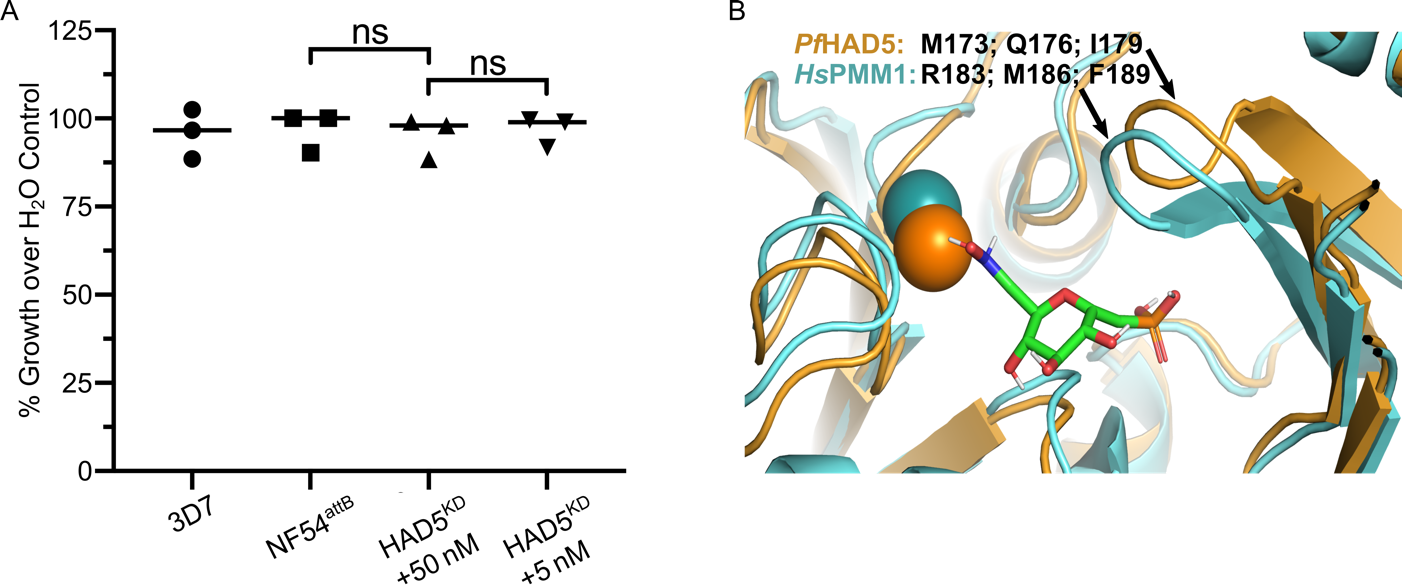


**Figure S9. Compound D9 does not inhibit parasites in culture​.** **(A)** Displayed is the percent growth after 72 hours of parasites (starting parasitemia ~1.2%) when treated with 100 µM compound D9, compared to vehicle control. The experiment was performed on wild type parasites of two different strains (3D7 and NF54), as well as HAD5^KD^ parasites ±aTc. Three independent experiments, each with technical replicates, were performed. Statistics were performed with an ordinary one-way ANOVA using Tukey's test for multiple comparisons, ns = not significant.​ **(B)** Shown is the D9 compound (green), computationally docked to *Pf*HAD5 crystal structure (orange), highlighting its location in the binding pocket, compared to subtle differences in the binding pocket of *Hs*PMM1 (cyan). Mg2+ ions are depicted from *Pf*HAD5 (dark orange) and *Hs*PMM1 (dark cyan) as spheres.​ Amino acid sequence variations within the active site loop are indicated (black arrows).

​

**Synthesis of phosphonate analogue of 6NHOH-G1CP (D9)**

**Scheme 1. Synthesis of phosphonate analogue of 6NHOH-G1CP (D9)**

The synthesis of α-D-glucose-1-phosphonate **D9** analogue commenced with the selective debenzylation-acetolysis (112) of the benzyl C-6 substituent of diethyl-C-(1-Deoxy 2,3,5,6-tetra-O-benzyl-α-D-gluctopyranosyl) methanephosphonate (**1**) using a solution of freshly fused ZnCl_2_ (5.2 equiv) in 1:5 HOAc–Ac2O in 98% yield. (Scheme 1). We observed that it was important to keep the temperature at 0 °C during the course of the reaction (30 min). The precursor phosphonate of **1** was synthesized according to our previously reported in three steps starting from 2,3,4,6-tetra-*O*-benzyl-D-glucopyranose (113). Conventional de-O-acetylation of **2** with potassium carbonate in methanol gave the alcohol **3** in high yield. Although deacetylation of the acetyl protecting group at C-6 in **2** was first attempted using a mixture 1:3:7 TEA-H_2_O-MeOH, but only poor yields of alcohol **2** was obtained mainly due to low solubility of **2**. Synthetic alcohol **3** was converted to the corresponding aldehyde **4** by using the Dess-Martin periodinane (DMP) in 98% yield (114). First we examined reaction with using 1 equivalent of DMP, but the yield was low, then we increased amounts of DMP to 1.5 and 2 equivalents. Our optimal result was obtained when we used 1.5 equivalents of DMP. Without further purification, condensation of aldehyde **4** with hydroxylamine hydrochloride in pyridine afforded the desired oxime **5** in 94% yield (114). Further reduction of **5** with sodium cyanoborohydride (NaBH_3_CN) under acidic condition provided the hydroxylamine **6** in 78% yield (114). Deprotection of both benzyl ether and ethyl ester groups was readily accomplished with an excess of iodotrimethylsilane (TMSI) (113, 115). For preparation of target phosphonate **D9**, different quantities of TMSI were examined. With 10 equivalents of TMSI compound **6,** was converted to the final product **D9** in 95% yield. It should be mentioned, that when more than 10 equivalents of TMSI was used, the reaction led to decomposition.

**((2R,3R,4S,5R,6S)-3,4,5-tris(benzyloxy)-6-((diethoxyphosphoryl)methyl)tetrahydro-2H-pyran-2-yl)methyl acetate (2).**

A solution of ZnCl_2_ (708 mg, 5.2 eq, 5.2 mmol) in Ac_2_O/AcOH (5:1, 12 mL) was cooled to 0 °C then a solution of **1** (674 mg, 1mmol) in Ac_2_O/AcOH (5:1, 12 mL) was added dropwise. The solution was stirred for overnight at room temperature under N_2_. After evaporation, the residue was suspended in water (20 mL) and extracted with DCM (40 mL x 2). The combined organic extracts were dried (MgSO_4_), filtered and concentrated. Purification by flash chromatography (Hexanes-EtOAc, 1:1) afforded compound **2** (660 mg, 98%) as a colorless liquid; Rf 0.50 (Hexanes-EtOAc, 1:3). ^1^H NMR (500 MHz, CDCl_3_), δ_H_ (ppm) 1.35 (6H, td, ^3^*J*_HH_ = 7.1 Hz, ^4^*J*_HP_ = 2.4 Hz, 2OCH_2_C*H*_3_), 2.06 (3H, s, CH_3_CO), 2.20-2.26 (2H, m, CH_2_-P), 3.57 (1H, t, ^3^*J*_HH_ = 9.1 Hz, H-4), 3.72-3.75 (2H, m, H-2, H-3), 3.80 (1H, dt, ^3^*J*_HH_ = 9.7 Hz, ^3^*J*_HH_ = 2.8 Hz, H-5), 4.10-4.16 (4H, m, 2OC*H*_2_CH_3_), 4.24 (1H, dd, ^2^*J*_HH_ = 12.0 Hz, ^3^*J*_HH_ = 2.1 Hz, H-6a or H-6b), 4.45 (1H, dd, ^2^*J*_HH_ = 12.0 Hz, ^3^*J*_HH_ = 3.6 Hz, H-6a or H-6b), 4.35-4.56 (1H, m, H-1), 4.58 (1H, d, ^2^*J*_HH_ = 10.9 Hz, CH_2_ of Bn), 4.68 (1H, d, ^2^*J*_HH_ = 11.5 Hz, CH_2_ of Bn), 4.71 (1H, d, ^2^*J*_HH_ = 11.5 Hz, CH_2_ of Bn), 4.81 (1H, d, ^2^*J*_HH_ = 11.5 Hz, CH_2_ of Bn), 4.87 (1H, d, ^2^*J*_HH_ = 10.9 Hz, CH_2_ of Bn), 4.94 (1H, d, ^2^*J*_HH_ = 10.9 Hz, CH_2_ of Bn), 7-28-7.36 (15H, m, 3Ph). ^13^C NMR (125 MHz, CDCl_3_), δ_C_ (ppm) 16.69 (d, ^3^*J*_CP_ = 3.7 Hz, 2OCH_2_*C*H_3_), 16.73 (d, ^3^*J*_CP_ = 3.7 Hz, 2OCH_2_*C*H_3_), 21.08 (*C*H_3_CO), 22.87 (d, ^1^*J*_CP_ = 144.0 Hz, CH_2_-P), 61.81 (d, ^2^*J*_CP_ = 6.4 Hz, O*C*H_2_CH_3_), 62.03 (d, ^2^*J*_CP_ = 6.1 Hz, O*C*H_2_CH_3_), 63.31 (CH_2_-6), 69.88 (d, ^2^*J*_CP_ = 5.1 Hz, C-1), 70.49 (C-5), 73.34, 75.14, 75.58 (3CH_2_ of Bn), 77.40 (C-4), 79.31 (d, ^3^*J*_CP_ = 12.7 Hz, C-2), 77.40 (C-3), 127.98, 128.14, 128.29, 128.67, 128.71, 137.95, 137.99, 138.53 (3Ph), 170.92 (C=O).^31^P NMR (121 MHz, CDCl_3_), δ_P_ (ppm) 28.88. HRMS (ESI, Positive Mode) calcd for C_34_H_43_NaO_9_P [M + Na] ^+^ 649.2537, found 649.2534.

**((2R,3R,4S,5R,6S)-3,4,5-tris(benzyloxy)-6-((diethoxyphosphoryl)methyl)tetrahydro-2H-pyran-2-yl)methyl acetate** **(3).**

To a solution of **2** (626 mg, 1 mmol) in MeOH (30 mL), K_2_CO_3_ (500mg, 3.6 mmol) was added at room temperature. The resulting solution was then stirred overnight. After evaporation, the residue was suspended in water (30 mL) and extracted with DCM (40 mL x 2). The combined organic extracts were dried (MgSO_4_), filtered and concentrated to provide the crude product (543 mg, 93%) as a colorless liquid which was used in subsequent reactions without further purification (Rf 0.22 (Hexanes-EtOAc, 1:3). ^1^H NMR (500 MHz, CDCl_3_), δ_H_ (ppm) 1.35 (6H, t, ^3^*J*_HH_ = 7.1 Hz, 2OCH_2_C*H*_3_), 1.78 (1H, brs, OH), 2.04-2.12 (1H, m, CH_2_-P), 2.32-2.41 (1H, m, CH_2_-P), 3.40 (1H, t, ^3^*J*_HH_ = 8.1 Hz, H-4), 3.60-3.67 (1H, m, H-2), 3.73-3.81 (3H, m, H-3, H-6a, H-6b), 3.85 (1H, td, ^3^*J*_HH_ = 8.3 Hz, ^3^*J*_HH_ = 2.6 Hz, H-5), 4.12-4.17 (4H, m, 2OC*H*_2_CH_3_), 4.43-4.45 (1H, m, H-1), 4.61-4.83 (6H, m, 3CH_2_ of Bn), 7-29-7.40 (15H, m, 3Ph). ^13^C NMR (125 MHz, CDCl_3_), δ_C_ (ppm) 16.62 (d, ^3^*J*_CP_ = 5.8 Hz, 2OCH_2_*C*H_3_), 24.38 (d, ^1^*J*_CP_ = 144.1 Hz, CH_2_-P), 61.97 (d, ^2^*J*_CP_ = 5.9 Hz, O*C*H_2_CH_3_), 62.00 (CH_2_-6), 62.25 (d, ^2^*J*_CP_ = 6.4 Hz, O*C*H_2_CH_3_), 68.66 (d, ^2^*J*_CP_ = 5.6 Hz, C-1), 73.42 (CH_2_ of Bn), 73.89 (C-5), 74.76, 75.14 (2CH_2_ of Bn), 77.42 (C-4), 78.88 (d, ^3^*J*_CP_ = 13.7 Hz, C-2), 80.79 (C-3), 127.95, 128.02, 128.09, 128.17, 128.19, 128.64, 128.67, 128.70, 137.98, 138.11, 138.47 (3Ph).^31^P NMR (121 MHz, CDCl_3_), δ_P_ (ppm) 29.79. HRMS (ESI, Positive Mode) calcd for C_32_H_41_NaO_8_P [M + Na] ^+^ 607.2431, found 607.2420.

**Diethyl(((2S,3R,4S,5S,6S)-3,4,5-tris(benzyloxy)-6-formyltetrahydro-2H-pyran-2-yl) methyl) phosphonate (4).**

To a solution of **3** (584 mg, 1 mmol) in anhydrous CH_2_Cl_2_ (30 mL), Dess-Martin periodinane (636 mg, 1.5 mmol, 1.5 equiv) was added at room temperature. The resulting solution was then stirred at same temperature for 4h under nitrogen. The solution was diluted CH_2_Cl_2_ (30 mL), and then saturated NaHCO_3_(aq) and saturated Na_2_S_2_O_3_(aq) were added to the reaction sequentially. The resulting mixture was stirred for another 30 min at room temperature. The organic layer was separated, and the aqueous layer was extracted with EtOAc. The combined organic extracts were dried (MgSO_4_), filtered and concentrated to provide the crude product (547 mg, 94%) as a colorless liquid which was used in subsequent reactions without further purification (Rf 0.30 (Hexanes-EtOAc, 1:3). ^1^H NMR (500 MHz, CDCl_3_), δ_H_ (ppm) 1.28-1.35 (6H, m, 2OCH_2_C*H*_3_), 2.11-2.19 (1H, m, CH_2_-P), 2.31-2.39 (1H, m, CH_2_-P), 3.52 (1H, dd, ^3^*J*_HH_ = 5.3 Hz, ^4^*J*_HP_ = 3.3 Hz, H-2), 3.76 (1H, t, ^3^*J*_HH_ = 5.3 Hz, H-3), 3.81 (1H, t, ^3^*J*_HH_ = 4.8 Hz, H-4), 4.09-4.18 (4H, m, 2OC*H*_2_CH_3_), 4.28 (1H, d, ^3^*J*_HH_ = 4.7 Hz, H-5), 4.46 (1H, d , ^2^*J*_HH_ = 11.6 Hz, CH_2_ of Bn), 4.53-4.57 (3H, m, H-1 and CH_2_ of Bn ), 4.62-4.70 (3H, m, CH_2_ of Bn ), 7-20-7.36 (15H, m, 3Ph), 9.83 (1H, s, CHO). ^31^P NMR (121 MHz, CDCl_3_), δ_P_ (ppm) 28.67.

**Diethyl (((2S,3R,4S,5R,6R)-3,4,5-tris(benzyloxy)-6-((E)-(hydroxyimino)methyl)tetrahydro-2H-pyran-2-yl) methyl)phosphonate (5).**

To a solution of **4** (582 mg, 1 mmol) in anhydrous pyridine (15 mL), hydroxylamine hydrochloride (104 mg, 1.5 mmol, 1.5 equiv) was added at room temperature. The resulting solution was then stirred at same temperature for 5h and then pyridine was removed at 50 °C under high vacuum. Water (30 mL) was added to the residue and the mixture was extracted with CH_2_Cl_2_ (30 mL x 2). The combined organic extracts were dried (MgSO_4_), filtered and concentrated. Purification by flash column chromatography (Hexanes-EtOAc, 1:2) afforded compound **5** as a mixture of E/Z isomers (561 mg, 94%) as a colorless liquid; Rf 0.41 (Hexanes-EtOAc, 1:3). ^1^H NMR (500 MHz, CDCl_3_), δ_H_ (ppm) 1.29-1.37 (6H, m, 2OCH_2_C*H*_3_), 2.07-2.50 (2H, m, CH_2_-P), 3.43-3.80 (3H, m, H-2, H-3, H-4), 4.06-4.19 (4H, m, 2OC*H*_2_CH_3_), 4.23-4.36 (1H, m, H-5), 3.48-4.55 (1H, m, H-1), 4.57-4.95 (6H, m, 3CH_2_ of Bn), 7-15-7.37 (16H, m, 3Ph, CH=N), 9.38 (1H, brs, OH). ^13^C NMR (125 MHz, CDCl_3_), δ_C_ (ppm) 16.52 (d, ^3^*J*_CP_ = 6.5 Hz, OCH_2_*C*H_3_), 16.60 (d, ^3^*J*_CP_ = 6.4 Hz, OCH_2_*C*H_3_), 22.36 (d, ^1^*J*_CP_ = 143.2 Hz, CH_2_-P), 62.05 (d, ^2^*J*_CP_ = 6.4 Hz, O*C*H_2_CH_3_), 62.20 (d, ^2^*J*_CP_ = 5.7 Hz, O*C*H_2_CH_3_), 70.10 (d, ^2^*J*_CP_ = 5.4 Hz, C-1), 71.31 (C-5), 73.39, 75.24, 75.43 (3CH_2_ of Bn), 78.81 (d, ^3^*J*_CP_ = 12.9 Hz, C-2), 80.13, 80.71 (C-3, C-4), 127.74, 127.82, 128.05, 128.12, 128.21, 128.51, 128.63, 128.70, 137.91, 138.01, 138.71 (3Ph), 148.14 (CH=N).^31^P NMR (121 MHz, CDCl_3_), δ_P_ (ppm) 28.96 (minor isomer), 30.46 (major isomer). HRMS (ESI, Positive Mode) calcd for C_32_H_40_NNaO_8_P [M + Na] ^+^ 620.2384, found 620.2391.

**Diethyl (((2S,3R,4S,5R,6R)-3,4,5-tris(benzyloxy)-6-((hydroxyamino)methyl)tetrahydro-2H-pyran-2-yl)methyl)phosphonate (6).**

To a solution of **5** (597 mg, 1 mmol) and NaBH_3_CN (125 mg, 2 mmol, 2 equiv) in MeOH (30 mL), a solution of HCl (6N in MeOH) was added dropwise at 0 °C until pH 1-3. The resulting solution was then stirred at room temperature for 10h. The reaction was quenched by the addition of saturated NaHCO_3_ (aq) and the mixture was extracted with EtOAc (40 mL x 2). The combined organic extracts were dried (MgSO_4_), filtered and concentrated. Purification by flash column chromatography (CH_2_Cl_2_-MeOH, 40:1) afforded compound **5** (467 mg, 78%) as a colorless liquid; Rf 0.50 (CH_2_Cl_2_-MeOH, 40:1). ^1^H NMR (500 MHz, CDCl_3_), δ_H_ (ppm) 1.35 (6H, td, ^3^*J*_HH_ = 7.1 Hz, ^4^*J*_HP_ = 1.7 Hz, 2OCH_2_C*H*_3_), 2.13-2.21 (1H, m, CH_2_-P), 2.33-2.42 (1H, m, CH_2_-P), 2.82 (1H, dd, ^2^*J*_HH_ = 13.6 Hz, ^3^*J*_HH_ = 9.5 Hz, H-6a or H-6b), 3.27 (1H, t, ^3^*J*_HH_ = 9.3 Hz, H-4), 3.40 (1H, dd, ^2^*J*_HH_ = 13.6 Hz, ^3^*J*_HH_ = 1.7 Hz, H-6a or H-6b), 3.70-3.79 (2H, m, H-2, H-3), 4.00 (1H, dd, ^3^*J*_HH_ = 9.5 Hz, ^3^*J*_HH_ = 1.7 Hz, H-5), 4.09-4.22 (4H, m, 2OC*H*_2_CH_3_), 4.35-4.41 (1H, m, H-1), 4.62 (1H, d, ^2^*J*_HH_ = 11.0 Hz, CH_2_ of Bn), 4.64 (1H, d, ^2^*J*_HH_ = 11.7 Hz, CH_2_ of Bn), 4.76 (1H, d, ^2^*J*_HH_ = 11.7 Hz, CH_2_ of Bn), 4.83 (1H, d, ^2^*J*_HH_ = 10.9 Hz, CH_2_ of Bn), 4.87 (1H, d, ^2^*J*_HH_ = 11.0 Hz, CH_2_ of Bn), 4.92 (1H, d, ^2^*J*_HH_ = 10.9 Hz, CH_2_ of Bn), 7-29-7.36 (15H, m, 3Ph). ^13^C NMR (125 MHz, CDCl_3_), δ_C_ (ppm) 16.61 (d, ^3^*J*_CP_ = 4.8 Hz, 2OCH_2_*C*H_3_), 16.64 (d, ^3^*J*_CP_ = 4.6 Hz, 2OCH_2_*C*H_3_), 24.16 (d, ^1^*J*_CP_ = 145.7 Hz, CH_2_-P), 55.59 (CH_2_-6), 61.96 (d, ^2^*J*_CP_ = 6.3 Hz, O*C*H_2_CH_3_), 62.68 (d, ^2^*J*_CP_ = 6.5 Hz, O*C*H_2_CH_3_),68.97 (C-5), 68.93 (d, ^2^*J*_CP_ = 5.6 Hz, C-1), 73.70, 75.20, 75.71 (3CH_2_ of Bn), 79.63 (d, ^3^*J*_CP_ = 13.4 Hz, C-2), 80.80 (C-4), 82.13 (C-3) 172.89, 128.00, 128.04, 128.07, 128.09, 128.17, 128.60, 128.71, 138.00, 138.07, 138.58 (3Ph).^31^P NMR (121 MHz, CDCl_3_), δ_P_ (ppm) 28.83. HRMS (ESI, Positive Mode) calcd for C_32_H_43_NO_8_P [M] ^+^ 600.2721, found 600.2700.

**(((2S,3R,4S,5S,6R)-3,4,5-Trihydroxy-6-((hydroxyamino)methyl)tetrahydro-2H-pyran-2-yl) methyl)phosphonic acid (D9).**

To a solution of **6** (180 mg, 0.3 mmol) in anhydrous CH_2_Cl_2_ (3 mL), iodotrimethylsilane (427 μL, 3.0 mmol, 10 equiv) was added dropwise at 0 °C under nitrogen. The resulting solution was then stirred at room temperature for 3h. The reaction was quenched by the addition of methanol. The

mixture was concentrated, dissolved in H_2_O (10 mL) and washed with diethyl ether (20mL x 5). The aqueous layer was lyophilized to afford the target **D9** as a colorless foam (78 mg, 95%). ^1^H NMR (500 MHz, D_2_O), δ_H_ (ppm) 2.01-2.06 (1H, m, CH_2_-P), 2.21-2.27 (1H, m, CH_2_-P), 3.31 (1H, t, ^3^*J*_HH_ = 9.5 Hz, H-4), 3.57 (1H, dd, ^2^*J*_HH_ = 13.6 Hz, ^3^*J*_HH_ = 10.0 Hz, H-6a or H-6b), 3.57-3.62 (2H, m, H-3, H-6a or H-6b), 3.70-3.75 (1H, m, H-2), 3.92 (1H, dd, ^3^*J*_HH_ = 9.8 Hz, ^3^*J*_HH_ = 2.3 Hz, H-5), 4.39-4.41 (1H, m, H-1). ^13^C NMR (125 MHz, D_2_O), δ_C_ (ppm) 23.29 (d, ^1^*J*_CP_ = 136.9 Hz, CH_2_-P), 52.50 (CH_2_-6), 66.28 (C-5), 70.55 (d, ^3^*J*_CP_ = 12.9 Hz, C-2), 71.73 (C-4), 72.26 (d, ^2^*J*_CP_ = 5.8 Hz, C-1), 72.60 (C-3). ^31^P NMR (121 MHz, D_2_O), δ_P_ (ppm) 24.50. HRMS (ESI, Positive Mode) calcd for C_7_H_17_NO_8_P [M+1]^+^ 274.0686, found 274.0694.


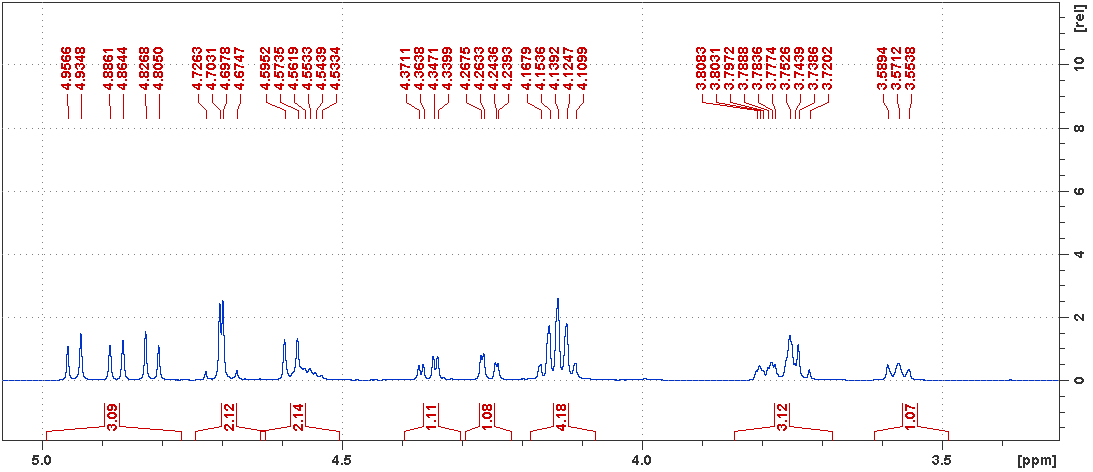

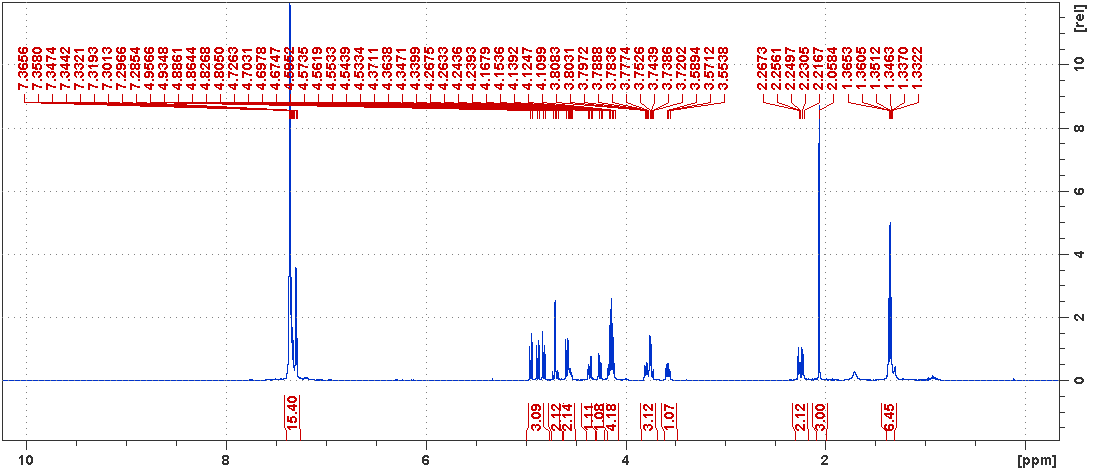


**Figure S10**. ^1^H NMR of compound **2**


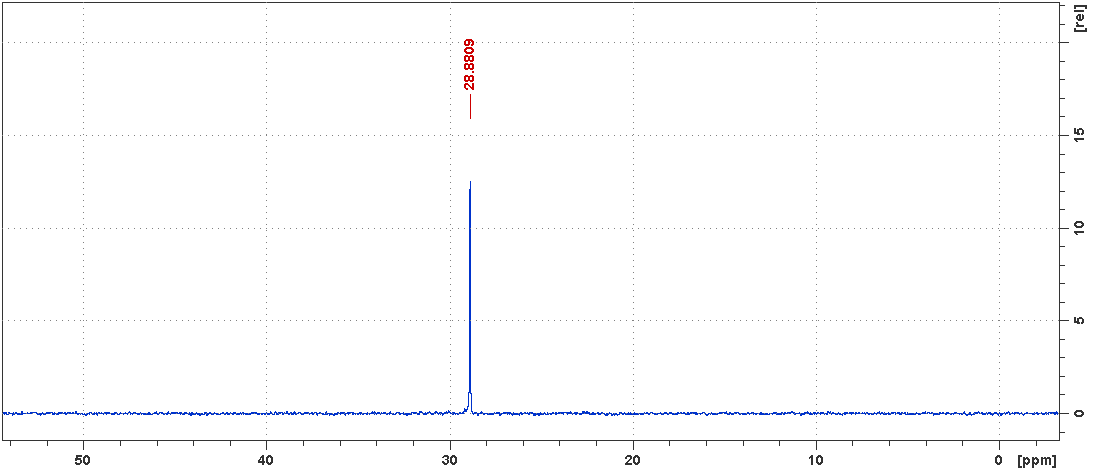


**Figure S11**. ^31^P NMR of compound **2**


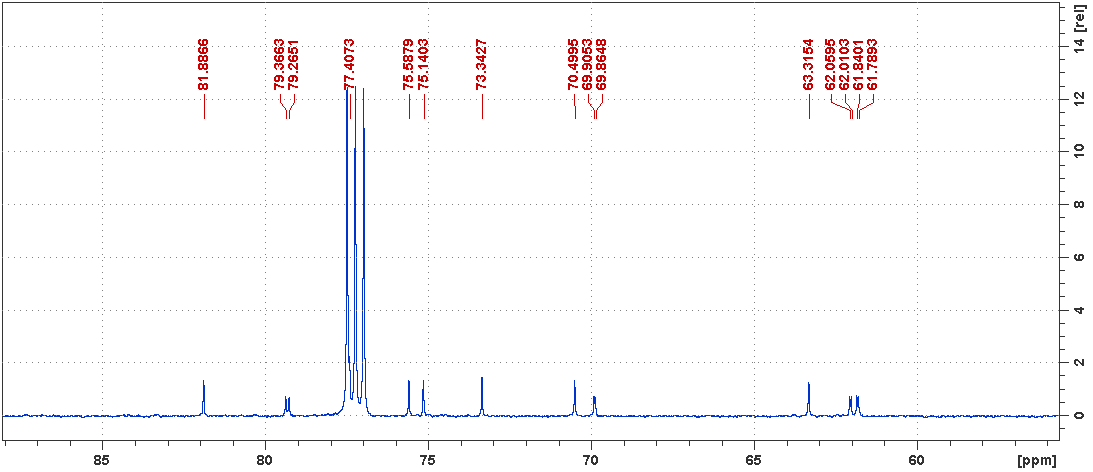

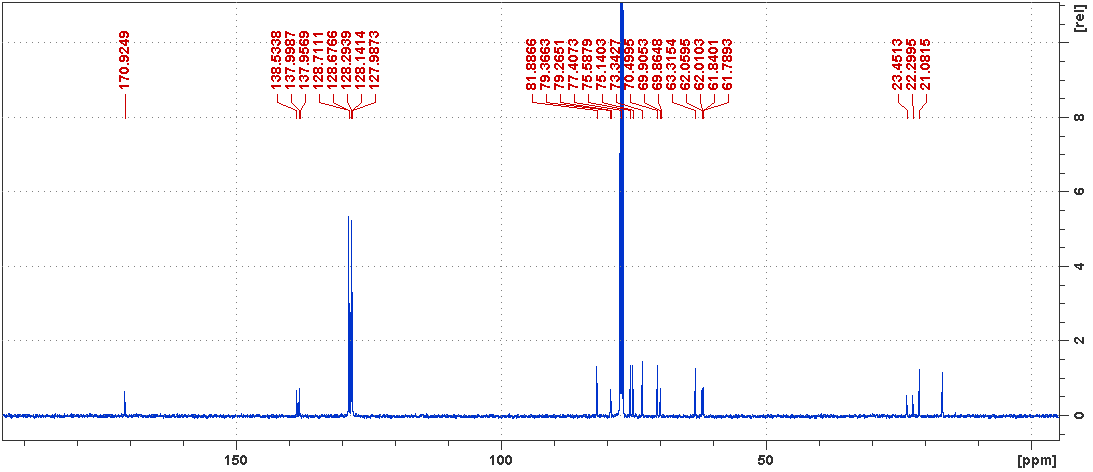


**Figure S12**. ^13^C NMR of compound **2**


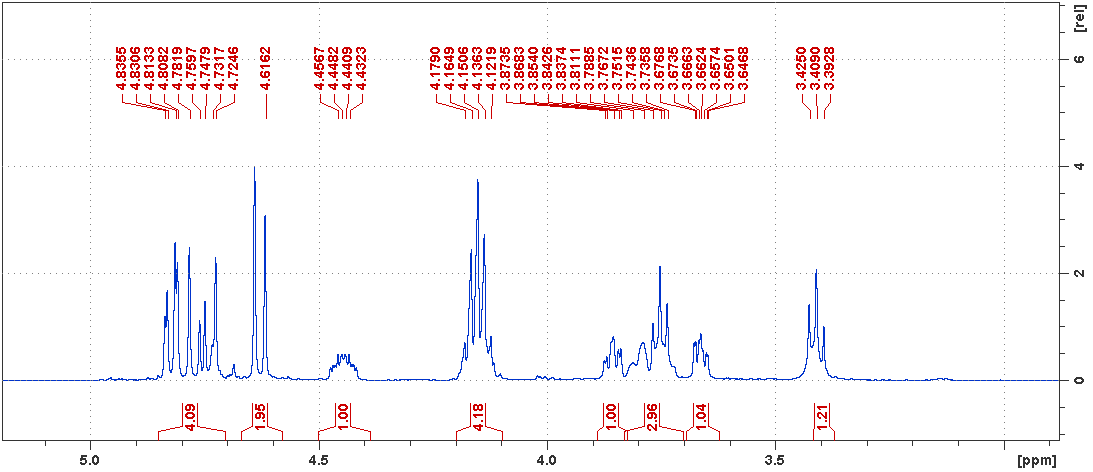

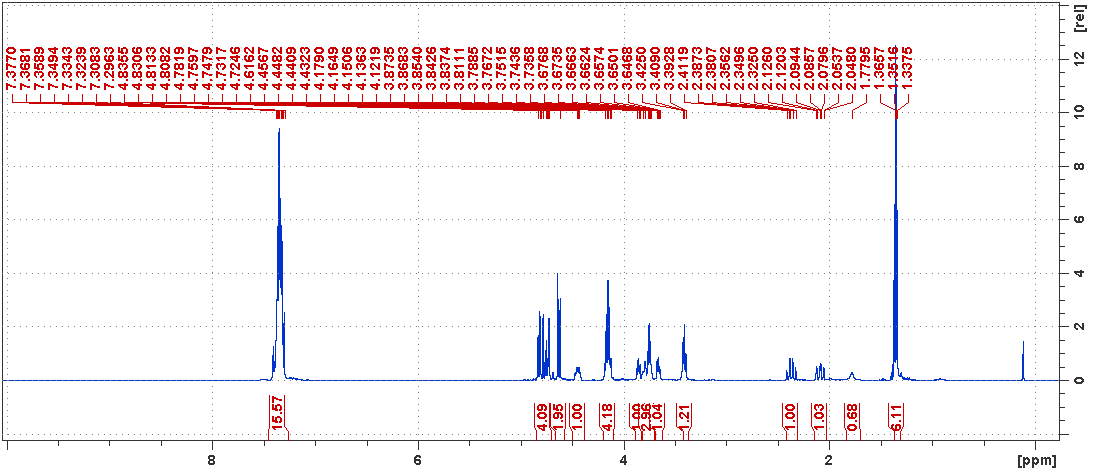


**Figure S13**. ^1^H NMR of compound **3**


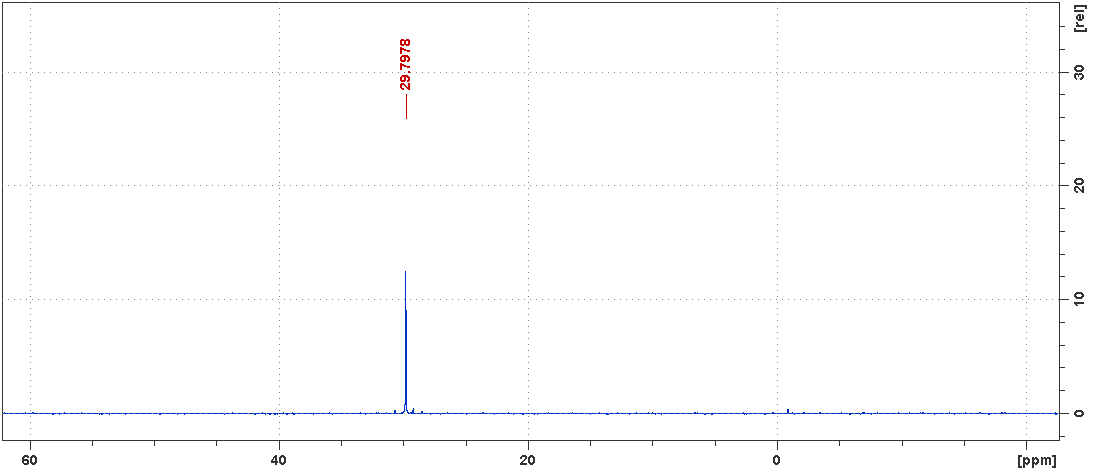


**Figure S14**. ^31^P NMR of compound **3**


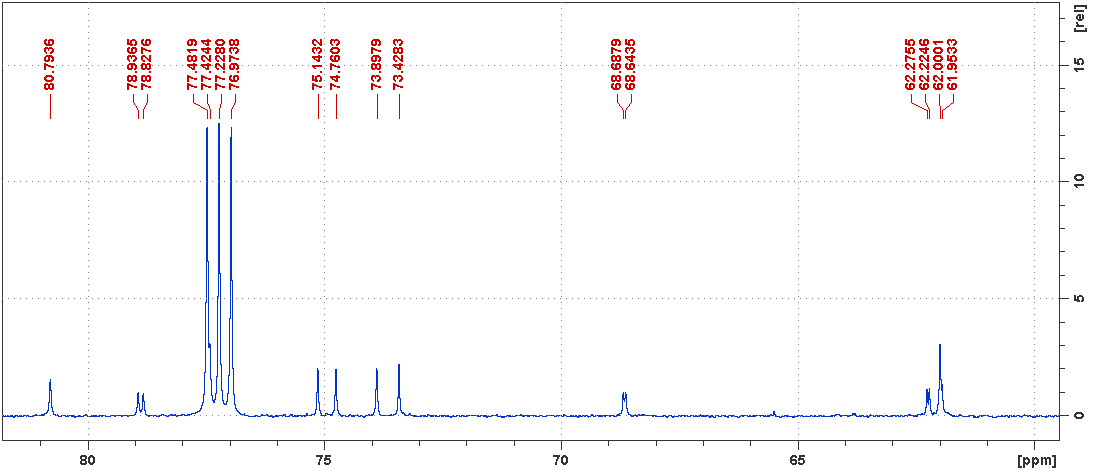

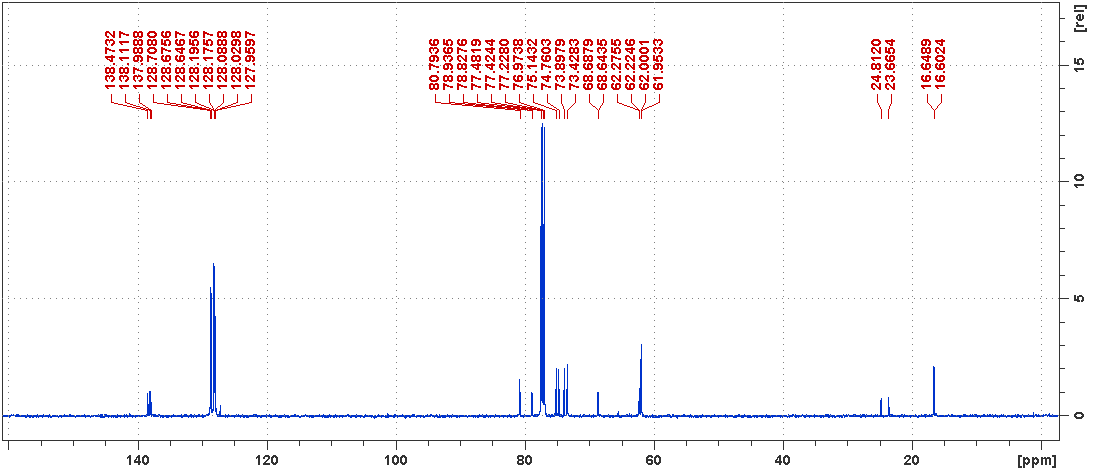


**Figure S15**. ^13^C NMR of compound **3**


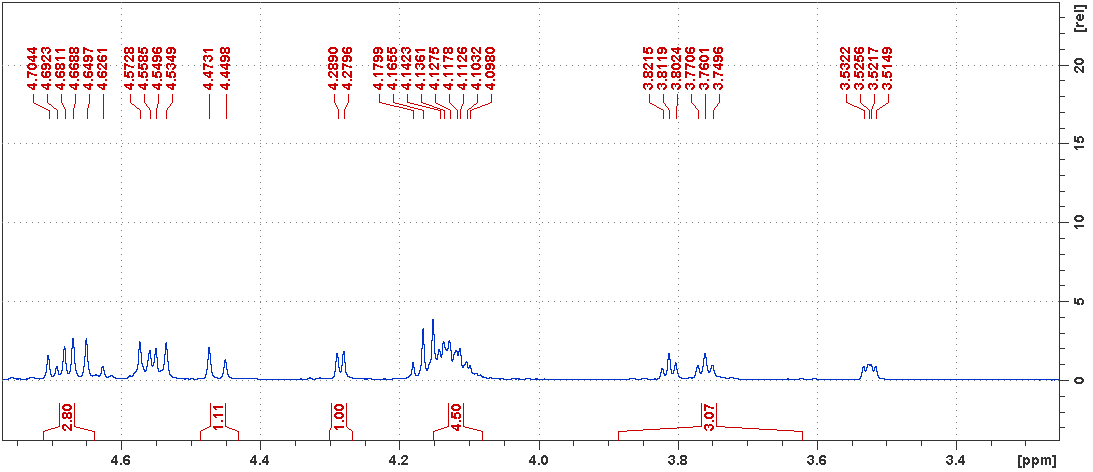

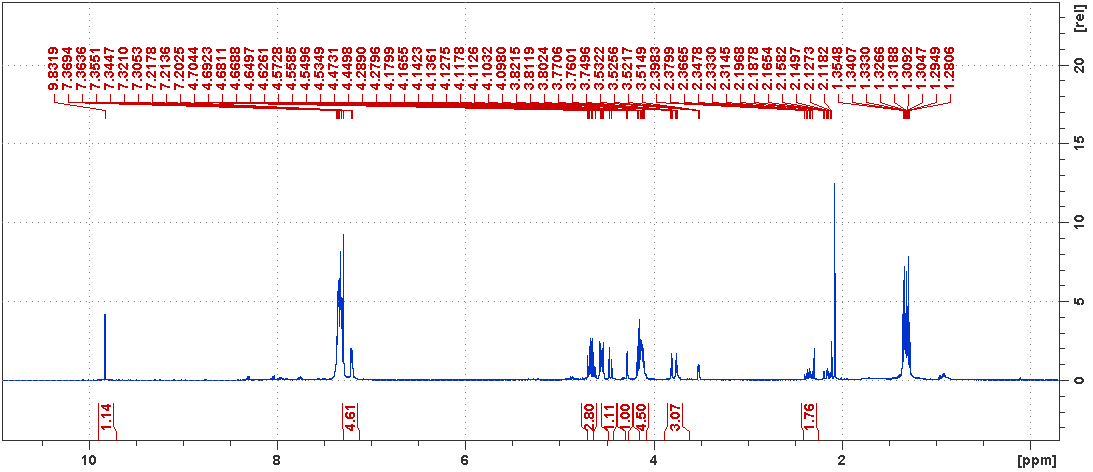


**Figure S16**. ^1^H NMR of compound **4**


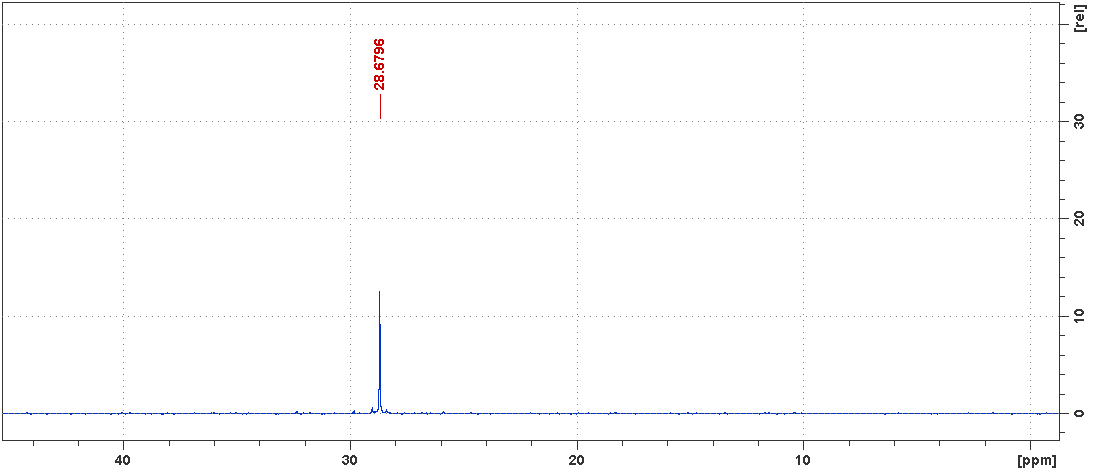


**Figure S17**. ^31^P NMR of compound **4**


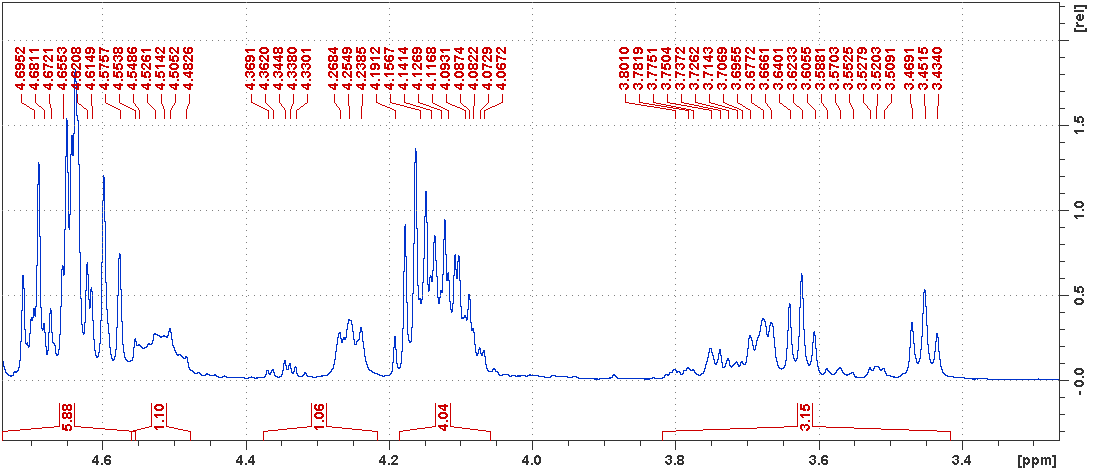

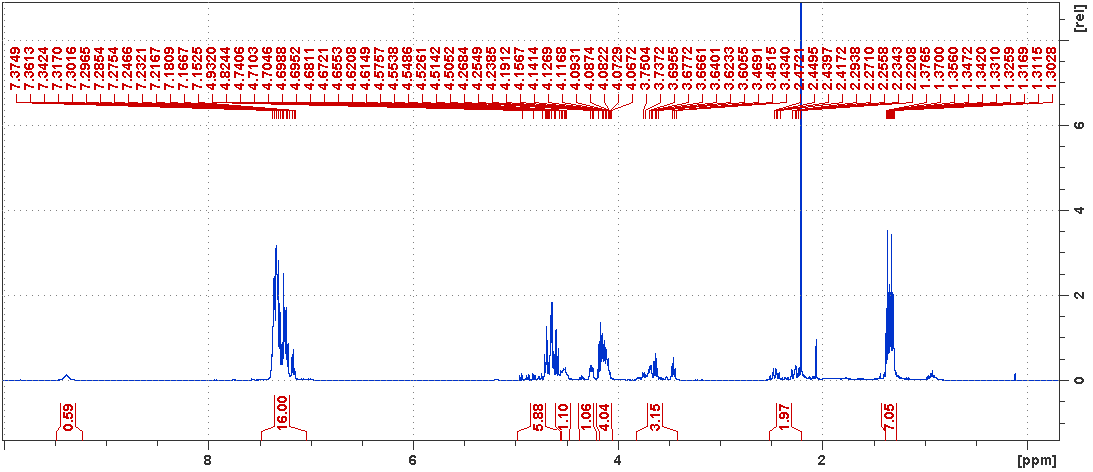


**Figure S18**. ^1^H NMR of compound **5**


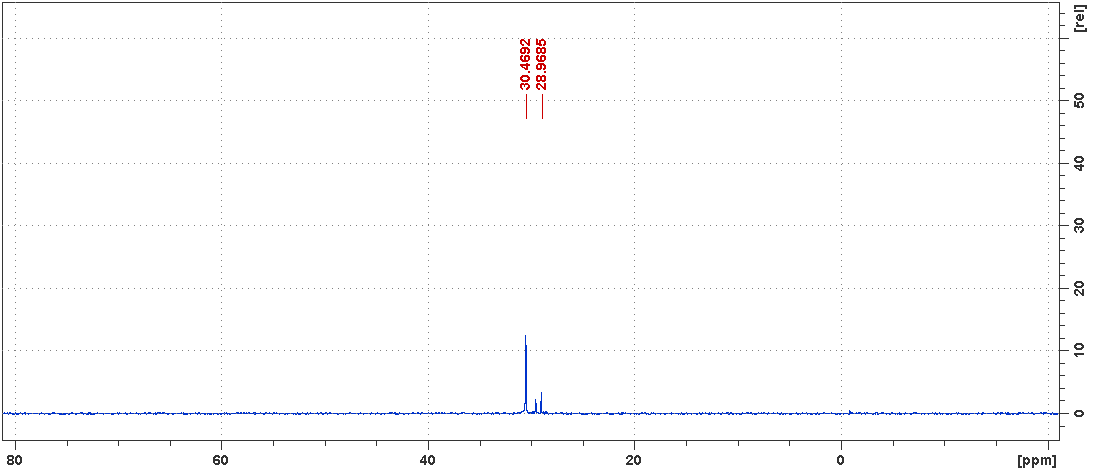


**Figure S19**. ^31^P NMR of compound **5**


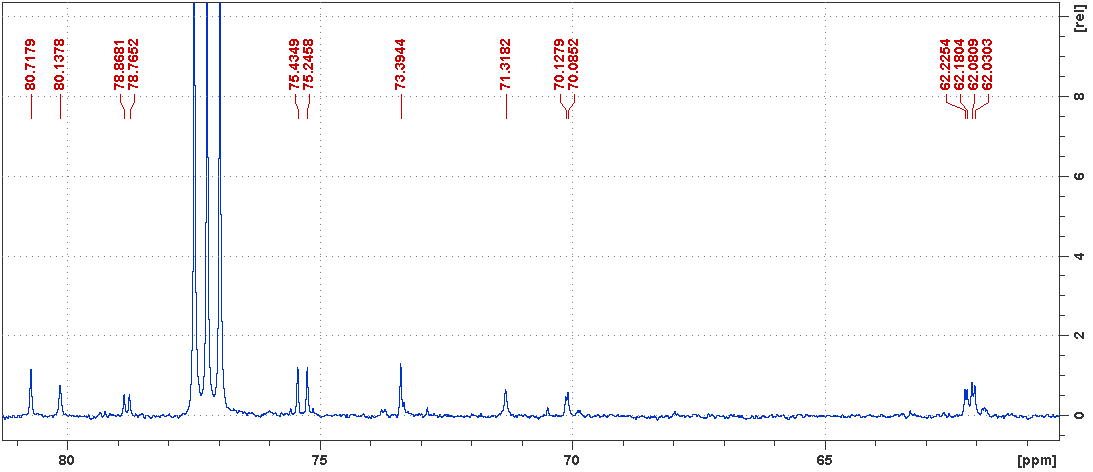

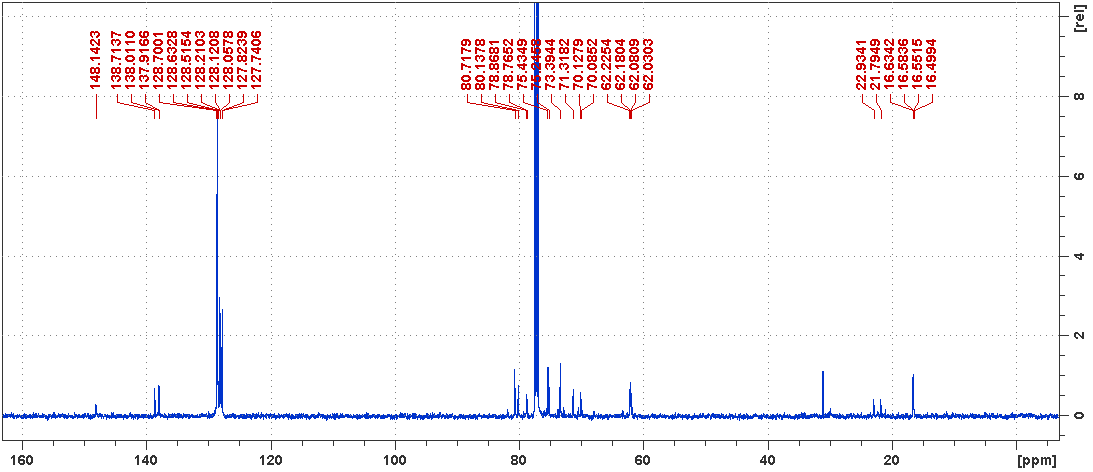


**Figure S20**. ^13^C NMR of compound **5**


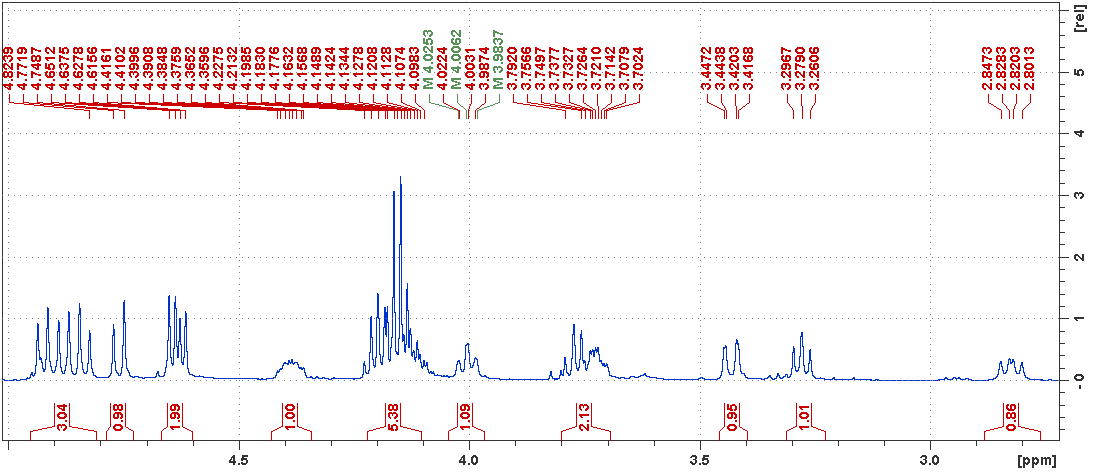

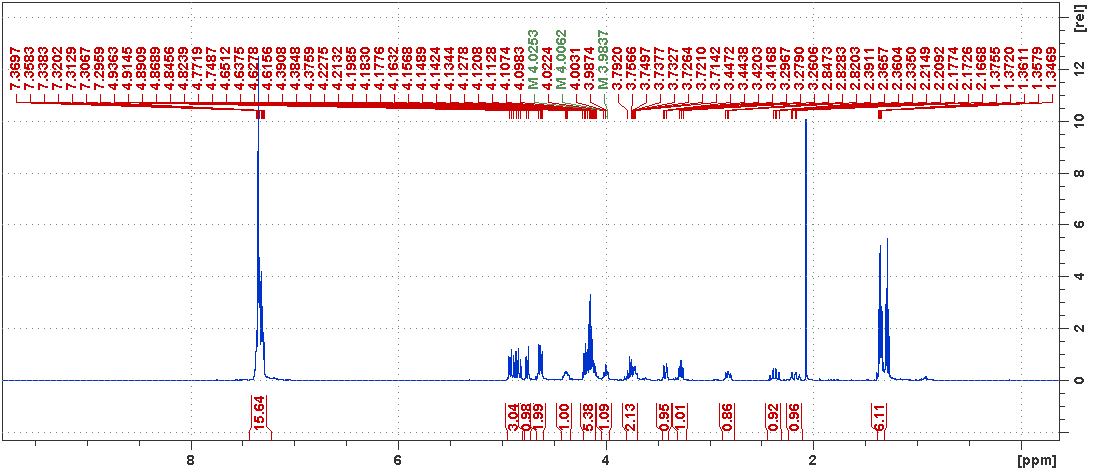


**Figure S21**. ^1^H NMR of compound **6**


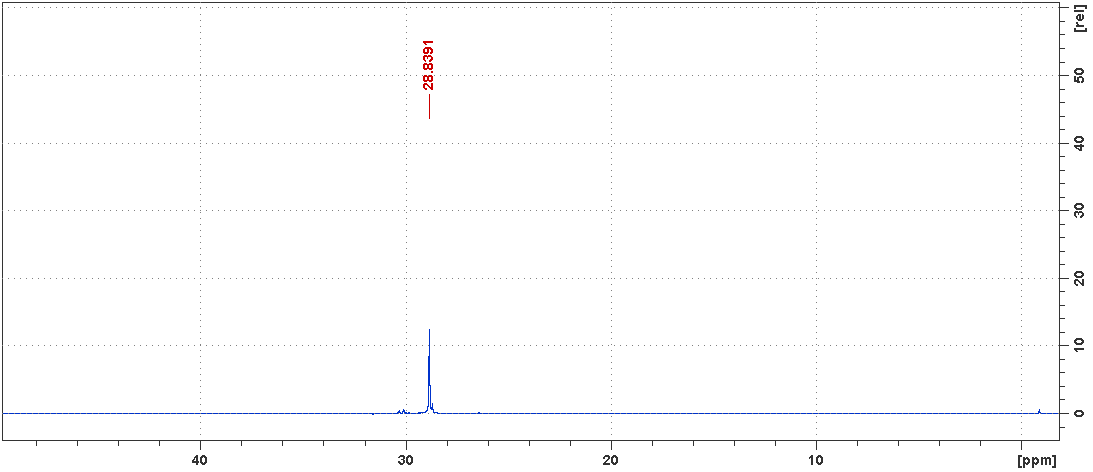


**Figure S22**. ^31^P NMR of compound **6**


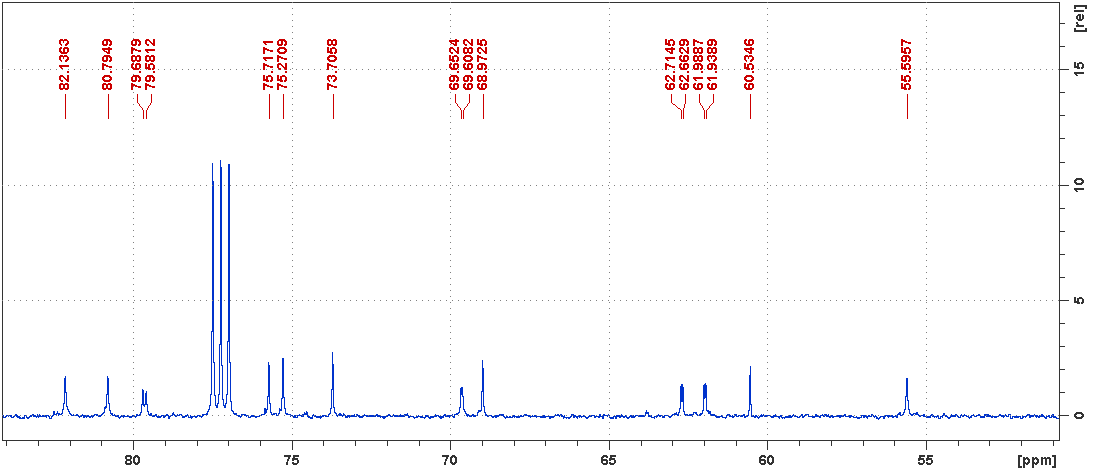

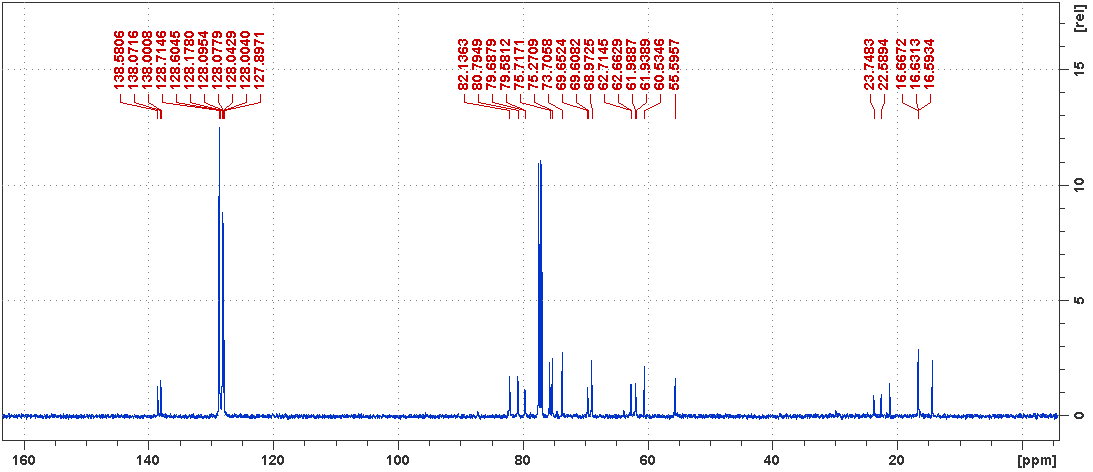


**Figure S23**. ^13^C NMR of compound **6**


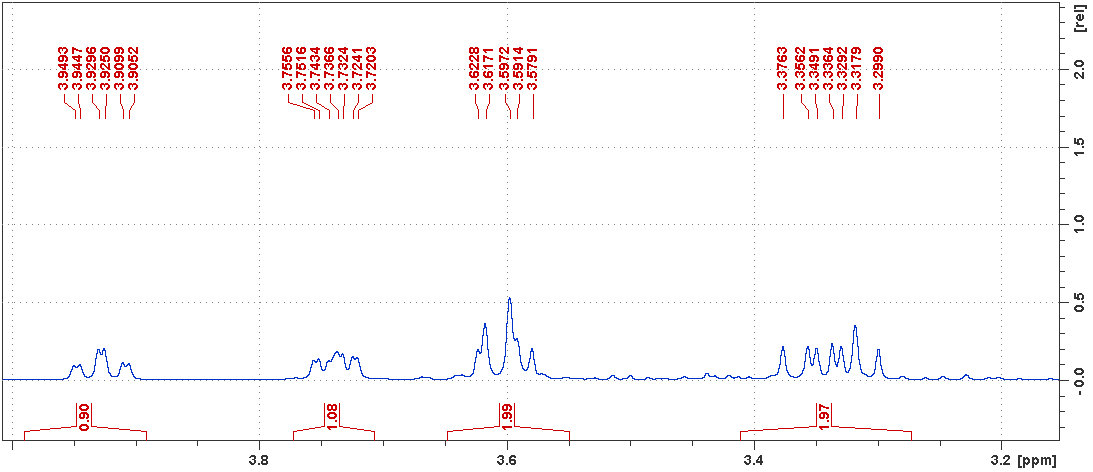

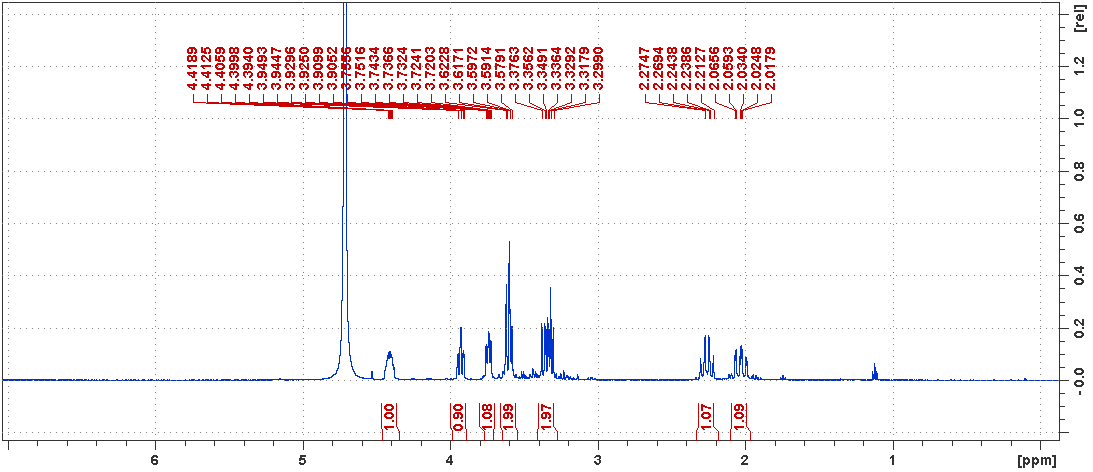


**Figure S24**. ^1^H NMR of compound **D9**


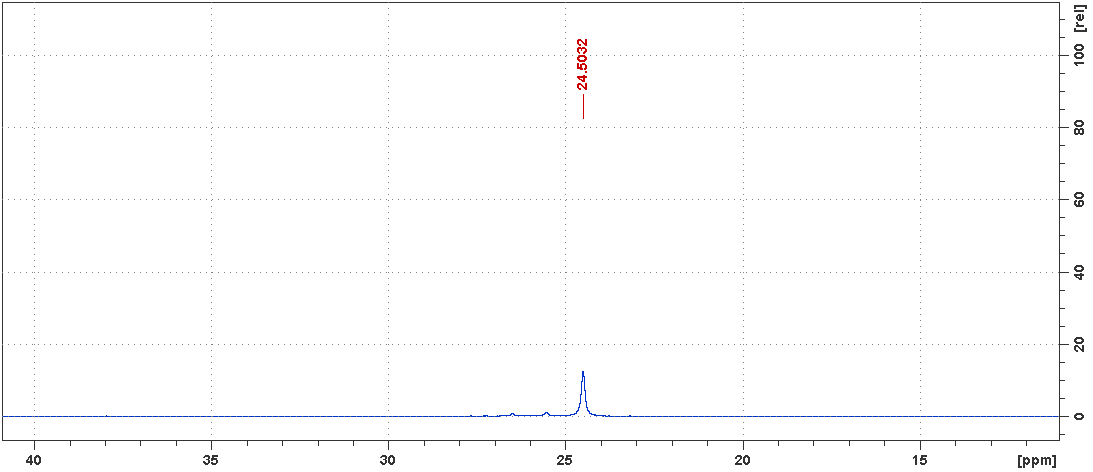


**Figure S25**. ^31^P NMR of compound **D9**


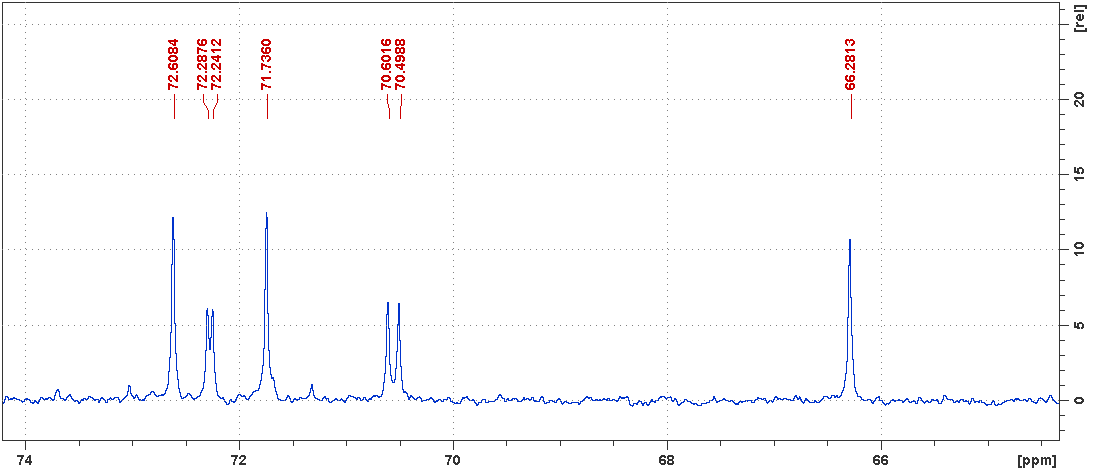

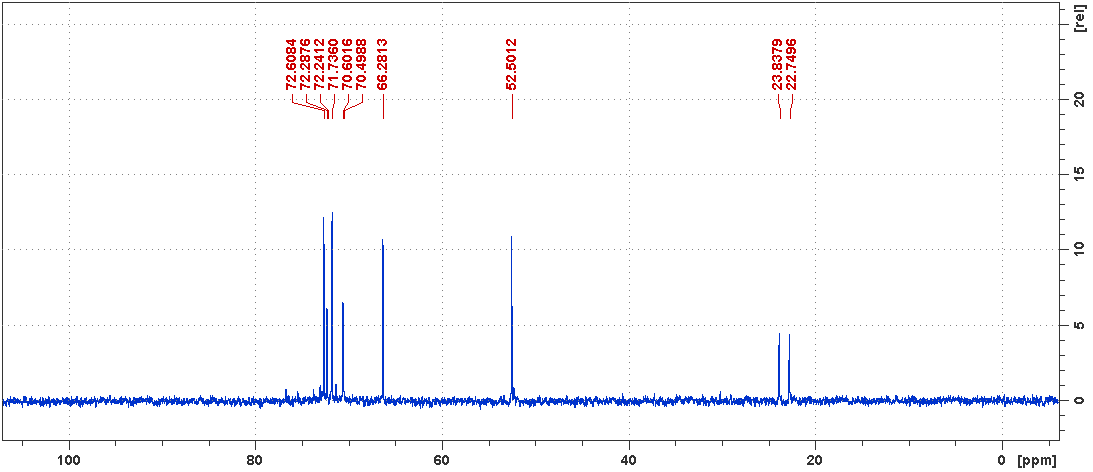


**Figure S26**. ^13^C NMR of compound **D9**

**References**

112. Lu, W., Navidpour, L., and Taylor, S. D. (2005) An expedient synthesis of benzyl 2,3,4-tri-O-benzyl-β-D- glucopyranoside and benzyl 2,3,4-tri-O-benzyl-β-D-mannopyranoside. *Carbohydr. Res.* **340**, 1213–1217

113. Beaton, S. A., Huestis, M. P., Sadeghi-Khomami, A., Thomas, N. R., and Jakeman, D. L. (2009) Enzyme-catalyzed synthesis of isosteric phosphono-analogues of sugar nucleotides. *Chem. Commun.* 10.1039/b808078j

114. Yuan, M. C., Yeh, T. K., Chen, C. T., Song, J. S., Huang, Y. C., Hsieh, T. C., Huang, C. Y., Huang, Y. L., Wang, M. H., Wu, S. H., Yao, C. H., Chao, Y. S., and Lee, J. C. (2018) Identification of an oxime-containing C-glucosylarene as a potential inhibitor of sodium-dependent glucose co-transporter 2. *Eur. J. Med. Chem.* **143**, 611–620

115. Nicotra, F., Ronchetti, F., and Russo, G. (1982) Stereospecific Synthesis of the Phosphono Analogues of α- and β-D-Glucose 1-Phosphate. *J. Org. Chem.* **47**, 4459–4462
